# Causal associations between dietary factors and colorectal cancer risk: a Mendelian randomization study

**DOI:** 10.3389/fnut.2024.1388732

**Published:** 2024-05-01

**Authors:** Xu Zhang, Zhimeng Wu, Xiangrui Wang, Binglong Xin, Ping Hu, Yan Yin, Shuixiang He, Mudan Ren

**Affiliations:** ^1^Department of Gastroenterology, The First Affiliated Hospital of Xi’an Jiaotong University, Xi’an, China; ^2^Shannxi Clinical Research Center of Digestive Disease (Cancer Division), Xi’an, China; ^3^Health Science Center, Xi’an Jiaotong University, Xi’an, China; ^4^Shanxi Institute of Science and Technology, Jincheng, China; ^5^Department of Surgery, Dangtu Central Health Center, Ma’anshan, China

**Keywords:** dietary factors, colorectal cancer, Mendelian randomization, causality, GWAS

## Abstract

**Background:**

Previous epidemiological studies have found a link between colorectal cancer (CRC) and human dietary habits. However, the inherent limitations and inevitable confounding factors of the observational studies may lead to the inaccurate and doubtful results. The causality of dietary factors to CRC remains elusive.

**Methods:**

We conducted two-sample Mendelian randomization (MR) analyses utilizing the data sets from the IEU Open GWAS project. The exposure datasets included alcoholic drinks per week, processed meat intake, beef intake, poultry intake, oily fish intake, non-oily fish intake, lamb/mutton intake, pork intake, cheese intake, bread intake, tea intake, coffee intake, cooked vegetable intake, cereal intake, fresh fruit intake, salad/raw vegetable intake, and dried fruit intake. In our MR analyses, the inverse variance weighted (IVW) method was employed as the primary analytical approach. The weighted median, MR-Egger, weighted mode, and simple mode were also applied to quality control. Heterogeneity and pleiotropic analyses were implemented to replenish the accuracy of the results.

**Results:**

MR consequences revealed that alcoholic drinks per week [odds ratio (OR): 1.565, 95% confidence interval (CI): 1.068–2.293, *p* = 0.022], non-oily fish intake (OR: 0.286; 95% CI: 0.095–0.860; *p* = 0.026), fresh fruit intake (OR: 0.513; 95% CI: 0.273–0.964; *p* = 0.038), cereal intake (OR: 0.435; 95% CI: 0.253–0.476; *p* = 0.003) and dried fruit intake (OR: 0.522; 95% CI: 0.311–0.875; *p* = 0.014) was causally correlated with the risk of CRC. No other significant relationships were obtained. The sensitivity analyses proposed the absence of heterogeneity or pleiotropy, demonstrating the reliability of the MR results.

**Conclusion:**

This study indicated that alcoholic drinks were associated with an increased risk of CRC, while non-oily fish intake, fresh fruit intake, cereal intake, and dried fruit were associated with a decreased risk of CRC. This study also indicated that other dietary factors included in this research were not associated with CRC. The current study is the first to establish the link between comprehensive diet-related factors and CRC at the genetic level, offering novel clues for interpreting the genetic etiology of CRC and replenishing new perspectives for the clinical practice of gastrointestinal disease prevention.

## 1 Introduction

Colorectal carcinoma (CRC) is the third most commonly diagnosed cancer worldwide, accounting for 9.4% of cancer-related fatalities globally (1). CRC patients exhibit clinical manifestations, including bowel habits changes, occult or overt rectal bleeding, abdominal pain, and anemia. However, in the early phase, patients are primarily asymptomatic or exhibit minor symptoms like common bowel diseases. When their bodies present a series of perceptible abnormalities, the cancer has already progressed to an advanced stage, even metastasized. Localized CRC patients have a high 5-year survival rate, decreasing from approximately 90% for primary tumors to 14% for metastatic CRC (2). With the incidence increasing constantly worldwide (1), CRC poses a significant challenge to public health globally. Individuals affected by CRC, including the patients and their families, fall into physical as well as financial adversities that ensue (3). Furthermore, CRC patients face psychological distress, including anxiety and depression (4). Eventually, the prolonged physical and mental issues may worsen the quality of life of patients. In addition, this disease not only presents a severe threat to personal health but also consumes substantial social and medical resources and heavily burdens society and healthcare systems (5). Clarifying the pathogenesis and etiology, including potential risk and protective factors, has excellent significance for the clinical practice of disease prevention and management.

Although the cause of CRC is still unclear, several researches have revealed some risk factors functionally integrated in the progression of this gastrointestinal disease. The Global Burden of Diseases, Injuries, and Risk Factors Study (GBD) indicated that the incidence rates of CRC increased with age, particularly surging in individuals aged 50–54 years and older (6). Additionally, a genome-wide association study identified 155 high-confidence effector genes that were functionally related to CRC risk, such as ARHGEF4, GNA12, LRIG1, GAB1, CNIH2, etc. These genes have multiple functions and affect tumor biology through various biological processes, including proliferation, homeostasis, migration, cell adhesion, immunity, and microbial interactions (7). Previous studies also found that environmental risk factors, Sedentary behavior (RR: 1.30, 95% CI: 1.22–1.39) (8) and smoking (RR: 1.17; 95% CI: 1.15–1.20) (9), could potentially impact the risk of CRC. Notably, in the realm of diet and nutrition, many experimental and epidemiological studies have made significant findings. For instance, Diets low in milk or calcium have been identified as primary contributors to the CRC disability-adjusted life years (6). Moreover, it has been found that nutritional supplements, such as omega-3 and arginine supplementation, could also modify the risk of CRC development (10).

According to previous studies, alcohol intake (11), red meat intake, processed meat intake (12), vegetable intake, and fruit intake (13) were associated with the pathophysiology of CRC. The potential mechanism of these pathologies is complicated and may contain direct biological effects on epithelial cells, modifications in inflammation and immune reactions, and diet-induced regulation in the composition and abundance of human gut microbes (14).

Current observational and meta-analysis studies on dietary factors and CRC face inherent limitations. Firstly, the sample sizes are typically small, affecting the reliability of results. Additionally, potential confounders may interfere with the interpretation of findings. Due to these factors, it’s challenging for these studies to conclusively demonstrate the epidemiological link between dietary habits and CRC risk. Hence, more robust and high-quality evidence is necessary to bridge the existing research gap.

Since the relationship between dietary factors and CRC has not been explored by any genetic instruments, we hypothesized there was a causative association of CRC with dietary factors. Similar to randomized controlled trials, the Mendelian randomization (MR) study is a novel research method that uses single-nucleotide polymorphisms (SNPs) as instrumental variables (IVs) to infer causal relationships between risk factors and health outcomes (15). This research methodology draws upon Mendel’s second law of genetics. It involves categorizing the study cohort according to the presence of specific genetic variations and subsequently comparing the occurrence of outcomes across these categories (16). SNPs follow the principle of being randomly allocated during the process of meiosis. This helps to eliminate the influence of confounding factors and the possibility of reverse causation, as genetic variants exist before the onset of the disease (17, 18). Recent MR studies suggest that dietary habits have a significant effect on several cardiovascular diseases (16) and five major mental disorders (19). Through MR studies, more diet-related factors for various diseases could be investigated. Herein, we performed a two-sample MR design to investigate the possible association of CRC with dietary factors.

## 2 Materials and methods

### 2.1 Study design

**Figure 1:**
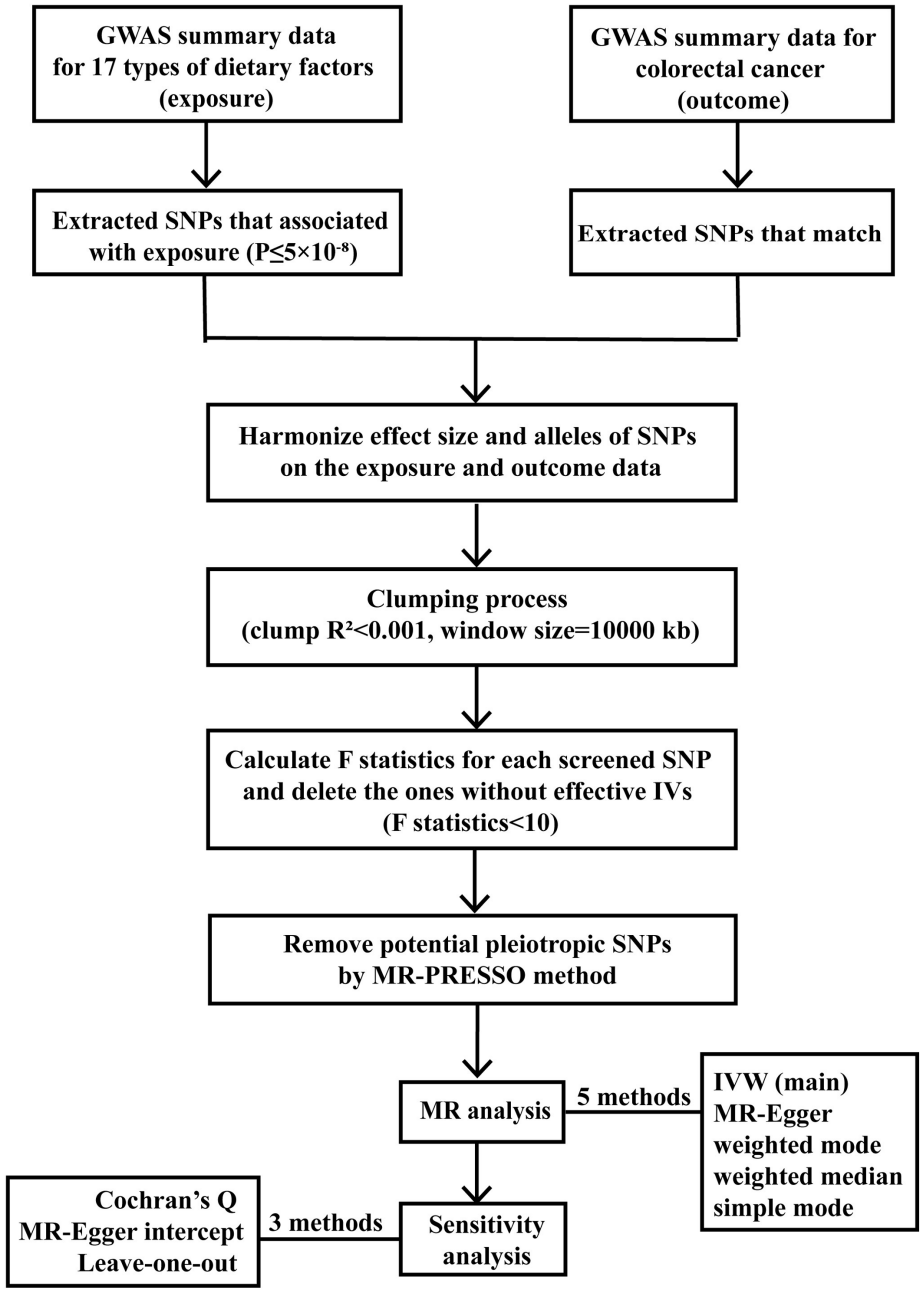
Study design and workflow.

A flowchart (Figure 1) presents our study design concisely, including the procedure of selecting IVs, conducting MR studies using five methods, and carrying out sensitivity analyses. To provide a better understanding of our study design, it’s important to detail the foundation of MR, which consists of three essential assumptions. The first assumption is that the SNPs employed as IVs are supposed to be closely related to exposure factors. The second assumption indicates that the screened IVs should not be associated with any confounding factors. The third assumption requires that the proposed genetic variants should impact the risk of the health outcome only via exposure we focused on Chen et al. (15). The three crucial assumptions guaranteed that the MR results would not be interfered with by other confounding factors, such as the population’s characteristics, environment, and socioeconomic status. Also, since the genetic variation explains the formation of the exposure part before the outcome, reverse causality can be eliminated, thus compensating for the limitations of traditional methods. The two-sample MR analysis was performed to identify the causal relationship between traits utilizing publicly available genetic datasets in several genome-wide association studies (GWAS).

### 2.2 Data sources

Dietary factors employed in our study covered drinks intake (alcoholic drinks per week, tea intake, and coffee intake), vegetable and fruit intake (salad/raw vegetable intake, cooked vegetable intake, fresh fruit intake and dried fruit intake), meat intake (pork intake, beef intake, lamb/mutton intake, poultry intake, oily fish intake, non-oily fish intake, and processed meat intake), staple diet intake (bread intake and cereal intake), and dairy product intake (cheese intake). These GWAS summary-level data were obtained from the UK Biobank by the IEU open GWAS project, supported by the MRC Integrative Epidemiology Unit (IEU) at the University of Bristol. The GWAS summary-level data of CRC was extracted from the European Bioinformatics Institute by the IEU open GWAS project. More relevant information about the original datasets is shown in Table 1 and Supplementary Table S1. All the data used in this work are publicly available and were obtained from studies with the consent and ethical approval of the relevant participants. As a result, this study did not require the ethical approval of an institutional review board.

**Table 1:** Information of the exposures and outcome datasets.

GWAS ID	Exposure/outcome	Identified SNPs	Participants included in the analysis	*F*-statistic
ieu-b-73	Alcoholic drinks per week	32	335,394 European-descent individuals	80.012
ukb-b-6324	Processed meat intake	23	461,981 European-descent individuals	38.536
ukb-b-8006	Poultry intake	7	461,900 European-descent individuals	32.539
ukb-b-2862	Beef intake	15	461,053 European-descent individuals	40.509
ukb-b-17627	Non-oily fish intake	11	460,880 European-descent individuals	44.802
ukb-b-2209	Oily fish intake	59	460,443 European-descent individuals	45.100
ukb-b-5460	Pork intake	14	460,162 European-descent individuals	37.686
ukb-b-14179	Lamb/mutton intake	31	460,006 European-descent individuals	39.797
ukb-b-11348	Bread intake	29	452,236 European-descent individuals	41.884
ukb-b-1489	Cheese intake	62	451,486 European-descent individuals	39.238
ukb-b-8089	Cooked vegetable intake	17	448,651 European-descent individuals	37.584
ukb-b-6066	Tea intake	40	447,485 European-descent individuals	61.576
ukb-b-3881	Fresh fruit intake	51	446,462 European-descent individuals	46.191
ukb-b-15926	Cereal intake	39	441,640 European-descent individuals	46.028
ukb-b-1996	Salad/raw vegetable intake	18	435,435 European-descent individuals	39.230
ukb-b-5237	Coffee intake	38	428,860 European-descent individuals	74.525
ukb-b-16576	Dried fruit intake	41	421,764 European-descent individuals	42.200
ebi-a-GCST012876	Colorectal cancer	NA	11,895 European-descent cases and 14,695 European-descent controls	NA

GWAS, Genome-Wide Association Studies; SNPs, single-nucleotide polymorphisms; N/A, not applicable.

### 2.3 Genetic variants

In order to meet the three assumptions of the MR analysis, the quality control steps below were applied to screen the related SNPs. We selected SNPs that are closely associated with various dietary factors. This selection was based on a genome-wide significant level (*p* < 5 × 10^−8^). We also performed the clumping process [distance window of 10,000 kb, linkage disequilibrium (LD) coefficient *r*^2^ < 0.001] (20). This step was crucial to avoid LD between SNPs and to ensure the independence of genetic variants. We selected the SNPs closely associated with various dietary factors at the significant level of genome-wide (*p* < 5 × 10^−8^) and conducted the clumping process [distance window 10,000 kb, linkage disequilibrium (LD) coefficient *r*^2^ < 0.001] to avoid LD between SNPs and ensure the independence of genetic variants (20). If no SNP was intensely related to any dietary factors found in the CRC database, proxy SNPs were allowed with a minimum LD *R*^2^ = 0.8 (21). Palindrome SNPs were reserved based on the threshold that the minor allele frequency (MAF) <0.3 (22). Notably, if the allele frequency contained in the details of an SNP is close to 0.5, we could hardly pinpoint the minor allele exactly, as there is sampling variance around the allele frequency. To enhance the accuracy of our study, we excluded such SNPs at the outset of MR analyses. In addition, to measure the power of the screened IVs and ensure their close relationships with exposures, we calculated the *F*-statistics and the proportion of variance interpreted (*R*^2^) for each SNP. Genetic variants (*F*-statistics <10) were generally considered as weak instruments, which should be removed from our MR analysis (23). Finally, MR-PRESSO tests were also employed to recognize potential horizontal pleiotropy, and the identified outliers would be ruled out to prevent the impact of pleiotropy (24).

### 2.4 Statistical analysis

We first performed an inverse variance weighted (IVW) test. This test is recognized for its strongest ability to determine causation (25). We applied it as the primary method to identify the causal effect between diet-related factors and CRC. We performed the inverse variance weighted (IVW) test, which possessed the most substantial ability to determine causation (25), as the significant method to detect the causal effect between diet-related factors and CRC. The evidence from the IVW method was complemented with the MR-Egger, weighted mode, weighted median, and simple mode. The conclusion would be more credible, stable, and precise when the consequences of these methods were consistent (26). For the IVW test and MR-Egger model, Cochran’s *Q* test was conducted to assess heterogeneity (27). Cochran’s *Q* test *p* < 0.05 indicated the existence of heterogeneity. Besides the MR-PRESSO test, as stated earlier, we also used the MR-Egger intercept test to detect directional pleiotropy. The absence of non-zero intercepts (*p* > 0.05) indicated that IVs did not affect CRC through other confounders (28). Leave-one-out analysis was applied to judge whether the causal link was affected by eliminating a particular SNP (29).

Statistical analysis was carried out with R software using the “TwoSampleMR” (20) package and “MR-PRESSO” (24). The significant threshold of the existence of causation is *p* < 0.05.

## 3 Results

### 3.1 Selection of instrumental variables

The causal associations of dietary factors with CRC were analyzed with 17 different exposures. The number of SNPs employed in our study ranged from 7 to 62. The *F*-statistics were greater than 10 for all the IVs (range: 32.539 to 80.012), suggesting that the selected IVs fulfilled the requirements of intense association with exposure. The amounts of European participants included in the exposure datasets ranged from 335,394 to 461,981. The outcome dataset covered 11,895 European-descent CRC cases and 14,695 European-descent controls. It was sourced from the European Bioinformatics Institute. Compared with the exposure datasets, there was little potential deviation in population stratification. More detailed information is presented in Table 1. Due to the non-significant conclusions of the MR-PRESSO global test (*p* > 0.05), no outlier was eliminated through MR-PRESSO.

### 3.2 MR analysis of dietary factors for CRC

**Figure 2:**
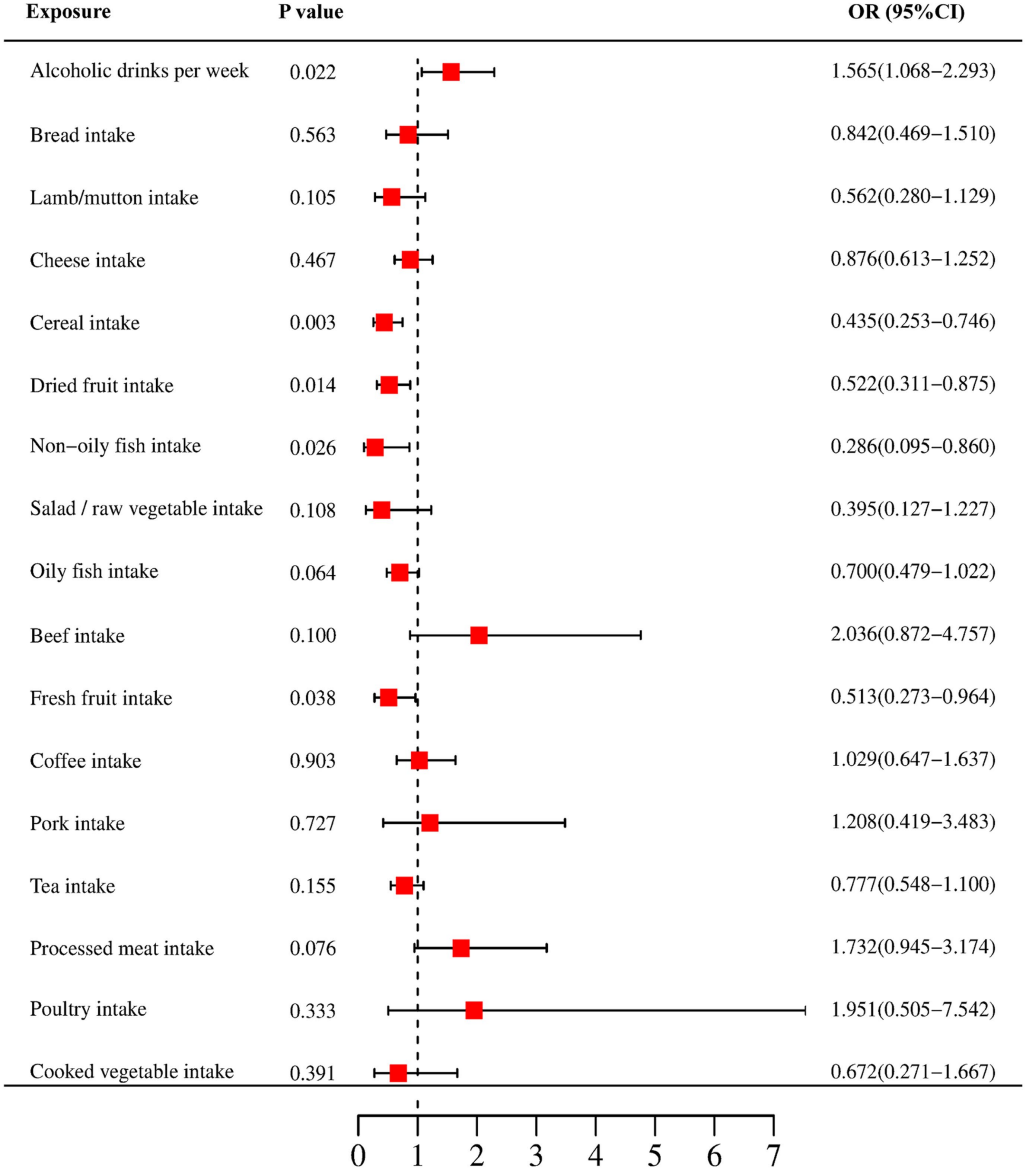
Forest plots of the MR results (IVW method) to present the causal associations between 17 dietary factors and CRC risk. OR, odds ratio; CI, confidence interval.

**Figure 3:**
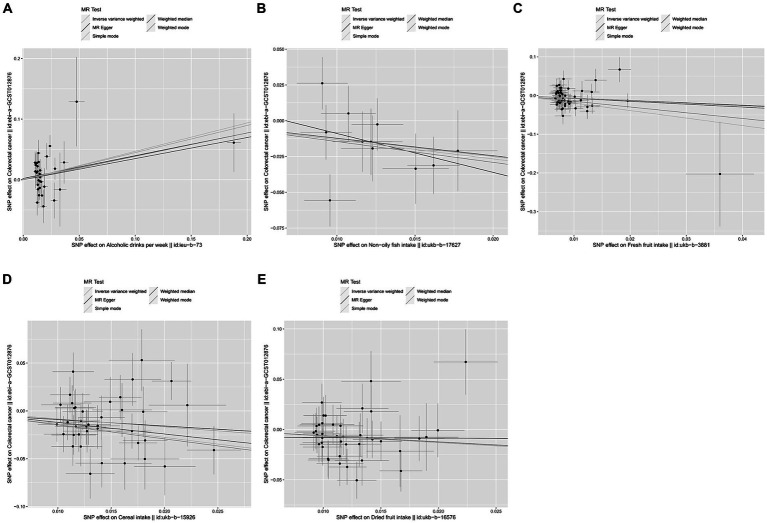
Scatter plots were used to visualize the causal effect between alcoholic drinks per week **(A)**, non-oily fish intake **(B)**, fresh fruit intake **(C)**, cereal intake **(D)**, dried fruit intake **(E)** and colorectal cancer. The *x*-axis shows the SNP effect and SE on dietary factors. The *y*-axis shows the SNP effect and SE on colorectal cancer. The regression lines for the inverse-variance weighted (IVW) method, the MR-Egger regression method, the weighted median, the weighted mode, and the simple mode are shown. The slope of each straight line indicates the magnitude of the causal association. SNP, single nucleotide polymorphism; SE, standard error.

In our study, a total of 5 causal relationships were discovered (*p* < 0.05 by IVW method). We identified that alcoholic drinks per week (OR: 1.565; 95% CI: 1.068–2.293; *p* = 0.022) was relevant to a higher risk of CRC. Non-oily fish intake (OR: 0.286; 95% CI: 0.095–0.860; *p* = 0.026), fresh fruit intake (OR: 0.513; 95% CI: 0.273–0.964; *p* = 0.038), cereal intake (OR: 0.435; 95% CI: 0.253–0.476; *p* = 0.003) and dried fruit intake (OR: 0.522; 95% CI: 0.311–0.875; *p* = 0.014) were all recognized as significantly protective factors. In addition, we have also reached positive conclusions in the weighted median model of cereal intake (OR: 0.299; 95% CI: 0.147–0.607; *p* = 0.001), oily fish intake (OR: 0.572; 95% CI: 0.332–0.985; *p* = 0.044) and the MR Egger model of cheese intake (OR: 5.490; 95% CI: 1.325–22.751; *p* = 0.022), although the IVW results of oily fish intake and cheese intake were non-significant. This study also found that other dietary factors were not associated with CRC. More specific analysis results are in Figures 2, 3 and Table 2.

**Table 2:** The results of Mendelian randomization analyses.

Exposure	MR method	nSNP	OR (95% CI)	*p*-val
Alcoholic drinks per week	MR Egger	32	1.455 (0.809–2.618)	0.220
	Weighted median	32	1.417 (0.886–2.265)	0.146
	Inverse variance weighted	32	1.565 (1.068–2.293)	0.022
	Simple mode	32	1.598 (0.433–5.89)	0.487
	Weighted mode	32	1.472 (0.889–2.435)	0.143
Processed meat intake	MR Egger	23	0.347 (0.016–7.337)	0.504
	Weighted median	23	1.300 (0.572–2.955)	0.531
	Inverse variance weighted	23	1.732 (0.945–3.174)	0.076
	Simple mode	23	1.181 (0.259–5.373)	0.832
	Weighted mode	23	1.160 (0.224–6.011)	0.861
Poultry intake	MR Egger	7	3.263 × 10^12^ (1.000 × 10^−3^–1.450 × 10^30^)	0.172
	Weighted median	7	1.540 (0.250–9.473)	0.641
	Inverse variance weighted	7	1.951 (0.505–7.542)	0.333
	Simple mode	7	0.941 (0.063–14.106)	0.966
	Weighted mode	7	0.941 (0.059–14.976)	0.967
Beef intake	MR Egger	15	3.686 (0.038–361.881)	0.587
	Weighted median	15	2.244 (0.736–6.839)	0.155
	Inverse variance weighted	15	2.036 (0.872–4.757)	0.100
	Simple mode	15	1.592 (0.174–14.579)	0.687
	Weighted mode	15	1.667 (0.208–13.325)	0.638
Non-oily fish intake	MR Egger	11	0.066 (0.000–15.012)	0.352
	Weighted median	11	0.295 (0.077–1.125)	0.074
	Inverse variance weighted	11	0.286 (0.095–0.860)	0.026
	Simple mode	11	0.263 (0.030–2.272)	0.253
	Weighted mode	11	0.233 (0.036–1.498)	0.156
Oily fish intake	MR Egger	59	0.349 (0.071–1.712)	0.200
	Weighted median	59	0.572 (0.332–0.985)	0.044
	Inverse variance weighted	59	0.700 (0.479–1.022)	0.064
	Simple mode	59	0.488 (0.152–1.572)	0.234
	Weighted mode	59	0.445 (0.146–1.355)	0.159
Pork intake	MR Egger	14	1.123 (0.001–1538.854)	0.975
	Weighted median	14	1.687 (0.407–6.987)	0.471
	Inverse variance weighted	14	1.208 (0.419–3.483)	0.727
	Simple mode	14	1.955 (0.182–21.001)	0.589
	Weighted mode	14	1.626 (0.12–22.026)	0.720
Lamb/mutton intake	MR Egger	31	1.660 (0.093–29.637)	0.733
	Weighted median	31	0.749 (0.293–1.919)	0.547
	Inverse variance weighted	31	0.562 (0.280–1.129)	0.105
	Simple mode	31	0.964 (0.158–5.879)	0.968
	Weighted mode	31	0.854 (0.152–4.815)	0.860
Bread intake	MR Egger	29	0.428 (0.024–7.732)	0.570
	Weighted median	29	1.006 (0.461–2.193)	0.989
	Inverse variance weighted	29	0.842 (0.469–1.510)	0.563
	Simple mode	29	0.893 (0.232–3.438)	0.870
	Weighted mode	29	1.066 (0.303–3.752)	0.922
Cheese intake	MR Egger	62	5.490 (1.325–22.751)	0.022
	Weighted median	62	0.956 (0.588–1.554)	0.855
	Inverse variance weighted	62	0.876 (0.613–1.252)	0.467
	Simple mode	62	0.872 (0.263–2.897)	0.824
	Weighted mode	62	0.976 (0.364–2.619)	0.962
Cooked vegetable intake	MR Egger	17	0.079 (0.000–1635.243)	0.624
	Weighted median	17	0.778 (0.217–2.788)	0.700
	Inverse variance weighted	17	0.672 (0.271–1.667)	0.391
	Simple mode	17	1.068 (0.083–13.724)	0.960
	Weighted mode	17	0.879 (0.086–8.949)	0.914
Tea intake	MR Egger	40	0.960 (0.425–2.168)	0.922
	Weighted median	40	0.793 (0.470–1.337)	0.385
	Inverse variance weighted	40	0.777 (0.548–1.100)	0.155
	Simple mode	40	0.955 (0.346–2.633)	0.929
	Weighted mode	40	0.922 (0.503–1.691)	0.795
Fresh fruit intake	MR Egger	51	0.539 (0.054–5.399)	0.602
	Weighted median	51	0.474 (0.182–1.236)	0.127
	Inverse variance weighted	51	0.513 (0.273–0.964)	0.038
	Simple mode	51	0.143 (0.019–1.099)	0.067
	Weighted mode	51	0.226 (0.045–1.132)	0.076
Cereal intake	MR Egger	39	0.509 (0.047–5.530)	0.583
	Weighted median	39	0.299 (0.147–0.607)	0.001
	Inverse variance weighted	39	0.435 (0.253–0.746)	0.003
	Simple mode	39	0.250 (0.054–1.156)	0.084
	Weighted mode	39	0.232 (0.058–0.923)	0.045
Salad/raw vegetable intake	MR Egger	18	4.179 (0.013–1388.484)	0.636
	Weighted median	18	0.677 (0.168–2.724)	0.583
	Inverse variance weighted	18	0.395 (0.127–1.227)	0.108
	Simple mode	18	1.814 (0.107–30.647)	0.685
	Weighted mode	18	2.346 (0.173–31.775)	0.530
Coffee intake	MR Egger	38	1.099 (0.420–2.876)	0.849
	Weighted median	38	1.080 (0.583–1.998)	0.808
	Inverse variance weighted	38	1.029 (0.647–1.637)	0.903
	Simple mode	38	1.202 (0.357–4.044)	0.768
	Weighted mode	38	1.043 (0.522–2.082)	0.906
Dried fruit intake	MR Egger	41	0.939 (0.088–10.052)	0.959
	Weighted median	41	0.540 (0.262–1.113)	0.095
	Inverse variance weighted	41	0.522 (0.311–0.875)	0.014
	Simple mode	41	0.544 (0.125–2.356)	0.420
	Weighted mode	41	0.544 (0.130–2.272)	0.409

*p*-val, *p*-value; SNP, single nucleotide polymorphism; OR, odds ratio; CI, confidence interval.

### 3.3 Sensitivity analysis

**Figure 4:**
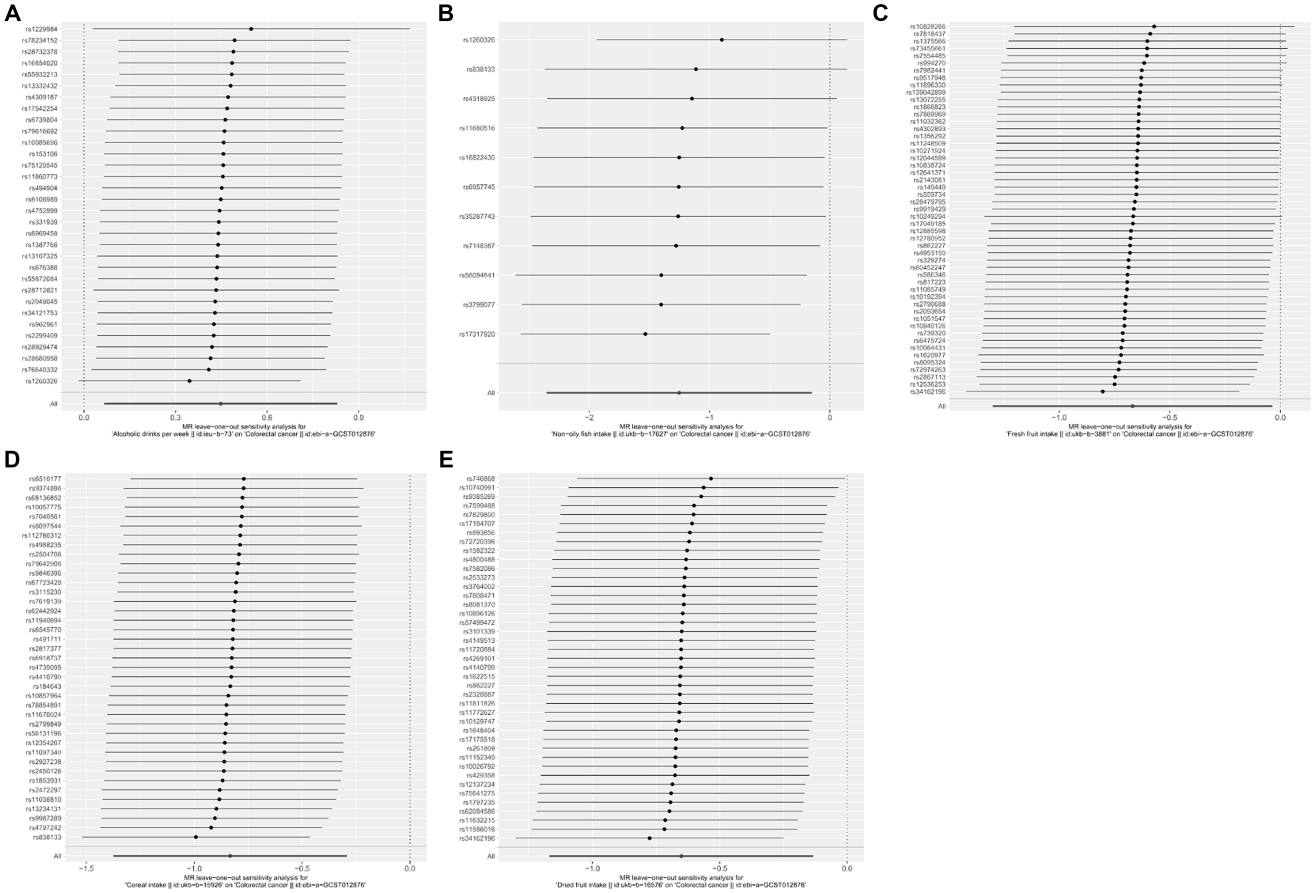
Forest plots of the “leave-one-out” sensitivity analyses to demonstrate the impact of individual SNPs on the results. The *x*-axis shows MR “leave-one-out” sensitivity analyses for alcoholic drinks per week **(A)**, non-oily fish intake **(B)**, fresh fruit intake **(C)**, cereal intake **(D)**, and dried fruit intake **(E)** on colorectal cancer. The *y*-axis shows the analyses for the effect of “leave-one-out” of SNPs on colorectal cancer. The black point on the bottom line of each panel indicates the IVW estimate using all SNPs. MR, Mendelian randomization; SNP, single nucleotide polymorphism.

Meanwhile, no heterogeneity was discovered in Cochran’s *Q* tests (*p* > 0.05 for all the consequences). MR-Egger intercept test indicated that except for the causality calculation between cheese intake and CRC, no statistically significant horizontal pleiotropy was observed in other remaining research (Figure 3 and Supplementary Table S3). Leave-one-out results suggested that no particular SNP could independently affect the MR positive conclusions (Figure 4). All the sensitivity analyses ensured the reliability of our results.

## 4 Discussion

We executed two-sample MR analyses utilizing large-scale GWAS summary statistics. These analyses observed genetic evidence for a causal association of CRC risk with 17 genetically predicted diet-related factors. Specifically, we noticed suggestive evidence that weekly alcoholic drinks may elevate CRC risk while a higher intake of non-oily fish, cereals, fresh fruit, and dried fruit may reduce risk. Apart from these five exposures, there was no evidence that other dietary factors affected CRC risk significantly. Clarifying these relationships had a vital impact on developing nutritional recommendations for CRC management and prevention.

The relationship between dietary factors and CRC remains controversial. Previously, some observational studies indicated that alcohol intake was a risk factor for colorectal cancer. For instance, a nested case-control study in South Asia revealed that current or former drinkers had a higher risk of CRC (OR: 5.4; 95% CI: 1.1–27.8; *p* = 0.043) (30). Similar conclusions were reported from other methods and regions (31, 32). However, a previous European MR study found no evidence of a pronounced relationship (OR: 1.60; 95% CI: 0.85–3.04; *p* = 0.146) (33). Whereas a total of 3 IVs representing weekly alcohol consumption were utilized, and only 0.2% of the genetic variation could be explained, which might lead to a weak statistical power and the absence of robustness. Our study, using 32 SNPs in total, preliminarily demonstrated that alcohol drinks per week was causally associated with about a 56.5% increase in the risk of CRC in European individuals. Some experimental evidence indicated that alcohol might result in the development of CRC by disrupting the composition of gut microbacteria. The possible acetaldehyde accumulation in the *Ruminococcus* and *Coriobacterium* located in the colorectum would contribute to mutagenesis and the enablement of carcinogenesis (34). Simultaneously, alcohol metabolites might trigger DNA-adduct formation, lipid peroxidation, and oxidative stress, leading to the initiation of cancer-promoting cascades (35). Additionally, an epigenetic analysis and a gene-alcohol interaction analysis revealed that alcohol consumption could affect DNA methylation by regulating the expression of the COLCA1/COLCA2 gene, which would also increase CRC risk (36). Further investigations are necessary to identify the role of alcohol intake in the genetic and metabolic effects of CRC.

The consequences are also inconsistent between fruit intake and the CRC risk. A European prospective investigation covering 2,819 incident CRC cases has shown that fruit consumption was inversely linked with CRC. The CRC risk was compared between the highest and the lowest EPIC-wide quintile of consumption over an 8.8-year follow-up (HR: 0.86; 95% CI: 0.75–1.00; *p* trend = 0.04) (37). Similarly, a cohort study on Chinese males obtained the same result (HR: 0.67; 95% CI: 0.48–0.95; *p* trend = 0.03) (38). On the contrary, a meta-analysis containing 16 cohort studies indicated the absence of significant association (39). Notably, the aforementioned conclusions might not be reliable due to the inherent drawbacks of the observational study design. Removing the underlying confounding factors and focusing on the fresh and dried fruit separately, our MR analyses suggested both fresh fruit (OR: 0.513; 95% CI: 0.273–0.964; *p* = 0.038) and dried fruit intake (OR: 0.522; 95% CI: 0.311–0.875; *p* = 0.014) were genetically correlated with a lower risk of CRC. The casual relationship may be attributable to several physiological mechanisms. Specifically, apigenin, a flavonoid that widely existed in fruits, targeted the K433 site of PKM2, thus restricted the glycolysis of HCT-8 and LS-174T cells, thereby serving the crucial function of anti-CRC *in vivo* and *in vitro* and markedly attenuating tumor growth in the meantime (40). Moreover, anthocyanins are phenolic pigments that give red and purple fruits their vivid colors. It has been demonstrated to protect against CRC by suppressing the activity and expression of DNA methyltransferase enzymes (DNMT1 and DNMT3B) and demethylating WNT upstream regulators (CDKN2A, SFRP2, SFRP5, and WIF1) (41). Further explorations were necessary to confirm the existence of the causality and investigate the concrete mechanism.

To date, the role of cereal intake in CRC has been widely studied, and a certain amount of epidemiological studies have yielded similar conclusions. A meta-analysis containing 7 European studies suggested a 10% decreased risk of CRC for each 10 g/day intake of cereal and more obvious reductions with higher intake (42). A prospective study of the UK Biobank deduced that intake of fiber from breakfast cereals was a statistically protective factor to CRC (HR: 0.86, 95% CI: 0.76–0.98, *p* trend = 0.005) with the multivariable model (43). Our results further confirmed the significant causal effect of cereal consumption (OR: 0.435; 95% CI: 0.253–0.746; *p* = 0.003) against the development of CRC. Mechanism studies reported that cereal foods could increase stool bulk, dilute fecal carcinogens, and decrease transit time. These procedures could offer the lining of the colorectum protective effects against carcinogens (44), which supported our discovery. Specifically, cereal foods’ regulatory effects on CRC development were mediated by activating AHR and GPCRs and inhibiting STAT3 phosphorylation (45). Analogically, other cereal components, including vitamins, phytoestrogens, and trace minerals, have also been associated with a lower risk of CRC (46). More underlying anticarcinogenic mechanisms of high levels of cereal intake could be investigated in the future.

In contrast, there is only a limited number of clinical studies focusing on non-oily fish and CRC. A large European cohort investigation observed an inverse association with CRC incidence (HR: 0.91; 95% CI: 0.83–1.00; *p* trend = 0.016) (47), which was compatible with our present study (OR: 0.286; 95% CI: 0.095–0.860, *p* = 0.0026). Additionally, pathophysiological evidence proposed that the ω-3 polyunsaturated fatty acids (PUFAs) contained in the fish might regulate eicosanoid metabolism (48). It was revealed that eicosapentaenoic acid (EPA), which is a type of ω-3 PUFAs, could lead to a decrease in the number and size of colorectal tumors by inhibiting COX-2, reducing β-catenin nuclear translocation and increasing apoptosis (49). ω-3 PUFAs could also promote a higher gut microbial diversity, thus ameliorating the body’s metabolic and immune functions and eventually reducing the CRC risk (34, 50). Subsequent high-quality analyses are required to deduce potential causalities and biological mechanisms.

Notably, some food of animal origins, such as dairy products and eggs, are susceptible to contamination by persistent organic pollutants (POPs), including polychlorinated dibenzo-p-dioxins (PCDDs), polychlorinated dibenzofurans (PCDFs) (51, 52), and polychlorinated biphenyls (PCBs) (53, 54). Long-term exposure to those POPs could damage the immune system and interfere with endocrine functions, thus causing a range of adverse health effects, especially cancer (51–54). Given that dietary intake is the primary route of exposure for humans, contaminated food of animal origin poses a significant risk to public health. Possible interventions, including vigilant monitoring, improved agricultural practices, regulatory enforcement, and public education, should be taken to reduce the risks associated with these contaminants.

There are multiple critical advantages of this work as follows: for all we know, this is the first work to elucidate the causal associations between CRC and diet-related factors by the two-sample MR method. This method addressed the debate of the prior epidemiologic studies and avoided the inherent deficiencies of previous traditional observational research, such as reverse causality and confounders. It also provided novel insights and methods for assessing the health benefits associated with dietary configurations. Secondly, benefiting from the large-scale GWAS database, the massive sample size of our analyses and the solid statistical evaluation effect of each IV (*F*-statistic >10) guaranteed the statistical validity of the current study. Moreover, we restricted the participants of this study to European-descent individuals, which minimized the potential bias induced by population stratification. Eventually, 5 MR methods and diverse sensitivity analyses were applied to assess the consistency of causal effects and obtained similar results, ensuring the robustness and stability of our discovery.

Some possible limitations in this study should also be considered. First, Mendel’s second law is not universally applicable to all genetic variants because not all genes determining traits are isolated independently. The inherent presence of developmental compensation bias also contributes to the potential inaccuracy of Mendelian randomization studies. Second, all analyses conducted in the current study were merely based on the European participants. Thus, it remained to be seen whether our results could be extrapolated to non-European populations. Third, due to the lack of classified population GWAS data for different sexes and ages, we could not execute a sex- or age-stratified analysis. Specifically, owing to the limited details provided by the original research, it was difficult to predict the generalizability of the study results across different exposure periods and levels. Analogically, diet-related information obtained from surveys may be prone to recall bias, which could possibly render our results unreliable. Additionally, given the complexity of dietary habits, we were unable to distinguish the impacts of diverse dietary combinations. Hence, it was challenging to identify the specific role of these interested dietary factors in the etiology and pathogenesis of CRC. Further investigation will focus on conducting more comprehensive studies to gather high-quality evidence regarding the idiographic mechanisms through which dietary factors affect CRC risk. This involves expanding the scope of research to include a broader range of dietary factors, identifying potential biomarkers that could help in understanding the effect of diet on CRC development, exploring genetic predispositions that may modify the impact of dietary factors, and longitudinal studies to track dietary habits over time and their direct correlation with CRC incidence.

## 5 Conclusion

Based on the GWAS summary data of CRC and European dietary habits, this study was implemented to identify the potential associations of colorectal cancer with 17 dietary factors using genetic instruments. The causal relationship between alcoholic drinks per week and an increased risk of CRC and the inverse causality of non-oily fish intake, cereal intake, fresh fruit, and dried fruit intake with CRC were determined by performing the two-sample MR analyses. The current study is the first to build the link between comprehensive diet-related factors and CRC at the genetic level, offering novel clues for interpreting the genetic etiology of CRC and replenishing new perspectives for managing gastrointestinal diseases. The result also prompts future explorations, including longitudinal studies and nutritional interventions, highlights the importance of interdisciplinary collaboration for clinical diagnostics, comprehensive patient care, and genetic counseling and education, and helps develop public health recommendations and tailored nutrition and prevention strategies.

## Data availability statement

The original contributions presented in the study are included in the article/Supplementary material, further inquiries can be directed to the corresponding authors.

## Author contributions

XZ: Formal analysis, Project administration, Conceptualization, Data curation, Investigation, Methodology, Resources, Software, Supervision, Validation, Visualization, Writing – original draft, Writing – review & editing. ZW: Conceptualization, Data curation, Formal analysis, Investigation, Methodology, Project administration, Resources, Software, Supervision, Validation, Visualization, Writing – original draft, Writing – review & editing. XW: Conceptualization, Data curation, Formal analysis, Investigation, Methodology, Resources, Software, Supervision, Validation, Visualization, Writing – original draft, Writing – review & editing. BX: Formal analysis, Investigation, Resources, Writing – review & editing, Data curation, Project administration, Validation, Writing – original draft. PH: Conceptualization, Investigation, Resources, Software, Writing – original draft, Formal analysis, Visualization. YY: Conceptualization, Supervision, Project administration, Writing – review & editing. SH: Conceptualization, Data curation, Formal analysis, Funding acquisition, Investigation, Software, Supervision, Validation, Visualization, Writing – original draft. MR: Methodology, Resources, Software, Validation, Writing – review & editing, Conceptualization, Funding acquisition, Supervision.
