# Supplementary material for: Causal associations between dietary factors and colorectal cancer risk: a Mendelian randomization study
Source: Front Nutr. 2024 May 1;11:1388732. doi: 10.3389/fnut.2024.1388732 (PMC11094808; doi:10.3389/fnut.2024.1388732)
Supplement: Supplementary file 1 [file Table_1.DOCX]

Supplementary Material

**Supplementary Table 1:**

**Detailed information about the exposure and outcome datasets**

**Supplementary Table 2:**

**Detailed information of each single nucleotide polymorphism selected in our study**

**Supplementary Table 3:**

**The result of sensitivity analyse**

**Supplementary Table 1** Detailed information about the exposure and outcome datasets.

| GWAS id | Exposure/outcome | definition | Source |
| --- | --- | --- | --- |
| ieu-b-73 | Alcoholic drinks per week | 1. Defined as the average number of drinks a participant reported drinking each week, aggregated across all types of alcohol. If a study recorded binned response ranges (e.g., 1-4 drinks per week, 5-10 drinks per week) we used the midpoint of the range. For example, if an individual reported 1-5 drinks per week, we assume they drank 2.5 drinks per week on average.  2. This was measured in a variety of ways.  a. In the past week, how many alcoholic beverages did you have?  b. Thinking about the past year, on the average how many drinks did you have each week?  3. This phenotype was left-anchored at 1 and log-transformed prior to analysis, in order to prevent outliers from having undue leverage on analyses. | N/A |
| ukb-b-16576 | Dried fruit intake | ACE touchscreen question "About how many pieces of DRIED fruit would you eat per DAY? (Count one prune, one dried apricot, 10 raisins as one piece; put '0' if you do not eat any)"  The following checks were performed:  If answer > 100 then rejected  If the participant activated the Help button they were shown the message:  Please provide an average considering your intake over the last year.  If you are unsure, please provide an estimate or select Do not know. | <https://biobank.ctsu.ox.ac.uk/crystal/field.cgi?id=1319> |
| ukb-b-6324 | Processed meat intake | ACE touchscreen question "How often do you eat processed meats (such as bacon, ham, sausages, meat pies, kebabs, burgers, chicken nuggets)?" If the participant activated the Help button they were shown the message: Please provide an average considering your intake over the last year. If you are unsure, please provide an estimate or select Do not know.  Options include:(1)never (2)once a week (3)less than once a week (4)2-4 times a week (5)5-6 time a week (6)once or more daily (7)do not know (8)prefer not to answer. | <https://biobank.ctsu.ox.ac.uk/crystal/field.cgi?id=1349> |
| ukb-b-8006 | Poultry intake | ACE touchscreen question "How often do you eat chicken, turkey or other poultry? (Do not count processed meats)" If the participant activated the Help button they were shown the message: Please provide an average considering your intake over the last year. If you are unsure, please provide an estimate or select Do not know.  Poultry include fowl (e.g. chicken, turkey, quail), waterfowl (e.g. duck, goose) and game (e.g. pheasant).  Options include:(1)never (2)once a week (3)less than once a week (4)2-4 times a week (5)5-6 time a week (6)once or more daily (7)do not know (8)prefer not to answer. | <https://biobank.ctsu.ox.ac.uk/crystal/field.cgi?id=1359> |
| ukb-b-2862 | Beef intake | ACE touchscreen question "How often do you eat beef? (Do not count processed meats)" If the participant activated the Help button they were shown the message: Please provide an average considering your intake over the last year.If you are unsure, please provide an estimate or select Do not know.  Options include:(1)never (2)once a week (3)less than once a week (4)2-4 times a week (5)5-6 time a week (6)once or more daily (7)do not know (8)prefer not to answer. | <https://biobank.ctsu.ox.ac.uk/crystal/field.cgi?id=1369> |
| ukb-b-17627 | Non-oily fish intake | ACE touchscreen question "How often do you eat other types of fish? (e.g. cod, tinned tuna, haddock)" If the participant activated the Help button they were shown the message: Please provide an average considering your intake over the last year.If you are unsure, please provide an estimate or select Do not know.  Options include:(1)never (2)once a week (3)less than once a week (4)2-4 times a week (5)5-6 time a week (6)once or more daily (7)do not know (8)prefer not to answer. | <https://biobank.ctsu.ox.ac.uk/crystal/field.cgi?id=1339> |
| ukb-b-2209 | Oily fish intake | ACE touchscreen question "How often do you eat oily fish? (e.g. sardines, salmon, mackerel, herring)" If the participant activated the Help button they were shown the message: Please provide an average considering your intake over the last year.If you are unsure, please provide an estimate or select Do not know.  Oily fish include:Salmon Anchovies, Trout Swordfish, Mackerel Bloater, Herring Cacha, Sardines Carp, Pilchards Hilsa, Kipper Jack fish, Eel Katla, Whitebait Orange roughy, Tuna (fresh only) Pangas, Sprats.  Options include:(1)never (2)once a week (3)less than once a week (4)2-4 times a week (5)5-6 time a week (6)once or more daily (7)do not know (8)prefer not to answer. | <https://biobank.ctsu.ox.ac.uk/crystal/field.cgi?id=1329> |
| ukb-b-5640 | Pork intake | ACE touchscreen question "How often do you eat pork? (Do not count processed meats such as bacon or ham)"  If the participant activated the Help button they were shown the message: Please provide an average considering your intake over the last year. If you are unsure, please provide an estimate or select Do not know.  Options include:(1)never (2)once a week (3)less than once a week (4)2-4 times a week (5)5-6 time a week (6)once or more daily (7)do not know (8)prefer not to answer. | <https://biobank.ctsu.ox.ac.uk/crystal/field.cgi?id=1389> |
| ukb-b-14179 | Lamb/mutton intake | ACE touchscreen question "How often do you eat lamb/mutton? (Do not count processed meats)"  If the participant activated the Help button they were shown the message: Please provide an average considering your intake over the last year. If you are unsure, please provide an estimate or select Do not know.  Options include:(1)never (2)once a week (3)less than once a week (4)2-4 times a week (5)5-6 time a week (6)once or more daily (7)do not know (8)prefer not to answer. | <https://biobank.ctsu.ox.ac.uk/crystal/field.cgi?id=1379> |
| ukb-b-11348 | Bread intake | ACE touchscreen question "How many slices of bread do you eat each WEEK?"  The following checks were performed:  If answer < 0 then rejected  If answer > 250 then rejected  If answer > 50 then participant asked to confirm  If the participant activated the Help button they were shown the message:  For other types of bread:  - one bread roll = 2 slices  - one pitta bread = 2 slices | <https://biobank.ctsu.ox.ac.uk/crystal/field.cgi?id=1438> |
| ukb-b-1489 | Cheese intake | ACE touchscreen question "How often do you eat cheese? (Include cheese in pizzas, quiches, cheese sauce etc)"  If the participant activated the Help button they were shown the message: Please provide an average considering your intake over the last year. If you are unsure, please provide an estimate or select Do not know.  Options include:(1)never (2)once a week (3)less than once a week (4)2-4 times a week (5)5-6 time a week (6)once or more daily (7)do not know (8)prefer not to answer. | <https://biobank.ctsu.ox.ac.uk/crystal/field.cgi?id=1408> |
| ukb-b-8089 | Cooked vegetable intake | ACE touchscreen question "On average how many heaped tablespoons of COOKED vegetables would you eat per DAY? (Do not include potatoes; put '0' if you do not eat any)"  The following checks were performed: If answer > 50 then rejected  If the participant activated the Help button they were shown the message:Please provide an average considering your intake over the last year. If you are unsure, please provide an estimate or select Do not know. If you have less than one tablespoon a day select Less than one. | <https://biobank.ctsu.ox.ac.uk/crystal/field.cgi?id=1289> |
| ukb-b-6066 | Tea intake | ACE touchscreen question "How many cups of tea do you drink each DAY? (Include black and green tea)"  The following checks were performed:  If answer < 0 then rejected  If answer > 99 then rejected  If answer > 20 then participant asked to confirm  If the participant activated the Help button they were shown the message: Please provide an average considering your intake over the last year. If you are unsure, please provide an estimate or select Do not know. | <https://biobank.ctsu.ox.ac.uk/crystal/field.cgi?id=1488> |
| ukb-b-3881 | Fresh fruit intake | ACE touchscreen question "About how many pieces of FRESH fruit would you eat per DAY? (Count one apple, one banana, 10 grapes etc as one piece; put '0' if you do not eat any)"  The following checks were performed:  If answer > 50 then rejected  If the participant activated the Help button they were shown the message:  Please provide an average considering your intake over the last year.  If you are unsure, please provide an estimate or select Do not know. | <https://biobank.ctsu.ox.ac.uk/crystal/field.cgi?id=1309> |
| ukb-b-15926 | Cereal intake | ACE touchscreen question "How many bowls of cereal do you eat a WEEK?"  The following checks were performed:  If answer < 0 then rejected  If answer > 99 then rejected  If answer > 14 then participant asked to confirm  If the participant activated the Help button they were shown the message:  Please provide an average considering your intake over the last year.  If you are unsure, please provide an estimate or select Do not know. | <https://biobank.ctsu.ox.ac.uk/crystal/field.cgi?id=1458> |
| ukb-b-1996 | Salad / raw vegetable intake | ACE touchscreen question "On average how many heaped tablespoons of SALAD or RAW vegetables would you eat per DAY? (Include lettuce, tomato in sandwiches; put '0' if you do not eat any)"  The following checks were performed:  If answer > 50 then rejected  If the participant activated the Help button they were shown the message:  Please provide an average considering your intake over the last year.  If you are unsure, please provide an estimate or select Do not know.  If you have less than one tablespoon a day select Less than one. | <https://biobank.ctsu.ox.ac.uk/crystal/field.cgi?id=1299> |
| ukb-b-5237 | Coffee intake | ACE touchscreen question "How many cups of coffee do you drink each DAY? (Include decaffeinated coffee)"  The following checks were performed:  If answer < 0 then rejected  If answer > 99 then rejected  If answer > 10 then participant asked to confirm  If the participant activated the Help button they were shown the message:  Please provide an average considering your intake over the last year.  If you are unsure, please provide an estimate or select Do not know. | <https://biobank.ctsu.ox.ac.uk/crystal/field.cgi?id=1498> |
| ukb-b-16576 | Dried fruit intake | ACE touchscreen question "About how many pieces of DRIED fruit would you eat per DAY? (Count one prune, one dried apricot, 10 raisins as one piece; put '0' if you do not eat any)"  The following checks were performed:  If answer > 100 then rejected  If the participant activated the Help button they were shown the message:  Please provide an average considering your intake over the last year.  If you are unsure, please provide an estimate or select Do not know. | <https://biobank.ctsu.ox.ac.uk/crystal/field.cgi?id=1319> |
| ebi-a-GCST012876 | Colorectal cancer | The Endpoint definition for colorectal cancer is in the ieu open GWAS project provided by Huyghe JR. | <https://gwas.mrcieu.ac.uk/datasets/ebi-a-GCST012876/> |

The data used in our study were mainly processed by the MRC Integrative Epidemiology Unit (IEU) at the University of Bristol from the UK Biobank and the European Bioinformatics Institute in 2018. The data of the UK Biobank and the European Bioinformatics Institute may also be partially updated. Therefore, the data from their official website and the data from IEU may not be completely consistent.More information on exposure and outcomes can be obtained on the website provided in the table. N/A: Not applicable

**Supplementary Table 2**  Detailed information of each single nucleotide polymorphism selected in our study.

| Exposure (Dietary factors) | SNP | Effect allele | Other allele | F-statistic | R2 | Exposure(Dietary factors) | | | Outcome (Colorectal cancer) | | |
| --- | --- | --- | --- | --- | --- | --- | --- | --- | --- | --- | --- |
|  |  |  |  |  |  | beta | se | pval | beta | se | pval |
| Alcoholic drinks per week | rs10085696 | G | A | 41.400 | 8.28E-05 | -0.0161 | 0.0025 | 1.24E-10 | 0.0034 | 0.0232 | 8.84E-01 |
| Alcoholic drinks per week | rs11860773 | C | T | 37.700 | 6.53E-05 | -0.0150 | 0.0024 | 8.35E-10 | 0.0027 | 0.0250 | 9.15E-01 |
| Alcoholic drinks per week | rs1229984 | C | T | 926.996 | 3.17E-03 | 0.1881 | 0.0062 | 1.00E-200 | 0.0612 | 0.0487 | 2.09E-01 |
| Alcoholic drinks per week | rs1260326 | C | T | 144.000 | 2.73E-04 | 0.0238 | 0.0020 | 3.33E-33 | 0.0557 | 0.0182 | 2.27E-03 |
| Alcoholic drinks per week | rs13107325 | T | C | 86.800 | 1.62E-04 | -0.0365 | 0.0039 | 1.23E-20 | -0.0286 | 0.0352 | 4.17E-01 |
| Alcoholic drinks per week | rs13332432 | G | C | 42.800 | 8.17E-05 | 0.0140 | 0.0021 | 5.94E-11 | -0.0257 | 0.0205 | 2.10E-01 |
| Alcoholic drinks per week | rs1387766 | A | G | 29.800 | 5.51E-05 | -0.0108 | 0.0020 | 4.79E-08 | -0.0138 | 0.0186 | 4.58E-01 |
| Alcoholic drinks per week | rs153106 | C | T | 48.300 | 8.96E-05 | -0.0136 | 0.0020 | 3.63E-12 | 0.0010 | 0.0184 | 9.58E-01 |
| Alcoholic drinks per week | rs16854020 | A | G | 38.700 | 7.25E-05 | 0.0181 | 0.0029 | 4.82E-10 | -0.0440 | 0.0277 | 1.13E-01 |
| Alcoholic drinks per week | rs17542254 | G | A | 37.500 | 6.49E-05 | 0.0131 | 0.0021 | 8.96E-10 | -0.0158 | 0.0203 | 4.36E-01 |
| Alcoholic drinks per week | rs2049045 | C | G | 30.200 | 5.81E-05 | -0.0138 | 0.0025 | 3.97E-08 | -0.0294 | 0.0231 | 2.03E-01 |
| Alcoholic drinks per week | rs2299409 | A | G | 29.800 | 5.57E-05 | -0.0106 | 0.0019 | 4.80E-08 | -0.0282 | 0.0181 | 1.19E-01 |
| Alcoholic drinks per week | rs28680958 | A | G | 32.900 | 6.54E-05 | -0.0136 | 0.0024 | 9.78E-09 | -0.0440 | 0.0217 | 4.27E-02 |
| Alcoholic drinks per week | rs28712821 | A | G | 206.001 | 3.87E-04 | 0.0283 | 0.0020 | 1.10E-46 | 0.0182 | 0.0184 | 3.22E-01 |
| Alcoholic drinks per week | rs28732378 | G | A | 58.300 | 1.11E-04 | -0.0167 | 0.0022 | 2.24E-14 | 0.0260 | 0.0203 | 2.00E-01 |
| Alcoholic drinks per week | rs28929474 | T | C | 44.600 | 6.89E-05 | -0.0477 | 0.0071 | 2.39E-11 | -0.1288 | 0.0739 | 8.12E-02 |
| Alcoholic drinks per week | rs331939 | A | G | 34.400 | 6.35E-05 | -0.0119 | 0.0020 | 4.50E-09 | -0.0116 | 0.0189 | 5.37E-01 |
| Alcoholic drinks per week | rs34121753 | G | A | 32.200 | 6.10E-05 | 0.0111 | 0.0020 | 1.39E-08 | 0.0244 | 0.0187 | 1.91E-01 |
| Alcoholic drinks per week | rs4309187 | C | A | 50.200 | 9.24E-05 | 0.0148 | 0.0021 | 1.37E-12 | -0.0135 | 0.0194 | 4.86E-01 |
| Alcoholic drinks per week | rs4752999 | T | C | 49.500 | 9.25E-05 | -0.0146 | 0.0021 | 2.03E-12 | -0.0097 | 0.0192 | 6.15E-01 |
| Alcoholic drinks per week | rs494904 | C | T | 59.200 | 1.11E-04 | 0.0151 | 0.0020 | 1.41E-14 | 0.0042 | 0.0185 | 8.19E-01 |
| Alcoholic drinks per week | rs55872084 | T | G | 31.500 | 5.53E-05 | 0.0127 | 0.0023 | 1.98E-08 | 0.0221 | 0.0212 | 2.97E-01 |
| Alcoholic drinks per week | rs55932213 | G | A | 31.700 | 6.53E-05 | 0.0125 | 0.0022 | 1.80E-08 | -0.0377 | 0.0212 | 7.56E-02 |
| Alcoholic drinks per week | rs6106989 | A | G | 30.200 | 5.55E-05 | 0.0109 | 0.0020 | 3.81E-08 | 0.0045 | 0.0186 | 8.10E-01 |
| Alcoholic drinks per week | rs6739804 | C | T | 38.800 | 7.55E-05 | -0.0130 | 0.0021 | 4.72E-10 | 0.0084 | 0.0192 | 6.62E-01 |
| Alcoholic drinks per week | rs676388 | C | T | 61.100 | 1.14E-04 | 0.0151 | 0.0019 | 5.49E-15 | 0.0155 | 0.0185 | 4.03E-01 |
| Alcoholic drinks per week | rs6969458 | A | G | 43.100 | 8.02E-05 | 0.0127 | 0.0019 | 5.20E-11 | 0.0135 | 0.0199 | 4.99E-01 |
| Alcoholic drinks per week | rs75120545 | T | C | 33.400 | 4.63E-05 | -0.0328 | 0.0057 | 7.59E-09 | 0.0162 | 0.0609 | 7.91E-01 |
| Alcoholic drinks per week | rs76640332 | A | G | 77.300 | 1.43E-04 | -0.0210 | 0.0024 | 1.47E-18 | -0.0385 | 0.0220 | 7.98E-02 |
| Alcoholic drinks per week | rs78234152 | A | G | 81.100 | 1.36E-04 | 0.0277 | 0.0031 | 2.18E-19 | -0.0343 | 0.0296 | 2.47E-01 |
| Alcoholic drinks per week | rs79616692 | C | G | 35.600 | 6.93E-05 | 0.0188 | 0.0032 | 2.38E-09 | -0.0114 | 0.0302 | 7.07E-01 |
| Alcoholic drinks per week | rs962961 | T | C | 35.300 | 6.56E-05 | -0.0122 | 0.0021 | 2.78E-09 | -0.0276 | 0.0192 | 1.52E-01 |
| Processed meat intake | rs10454812 | C | A | 33.626 | 7.36E-05 | -0.0200 | 0.0034 | 6.70E-09 | -0.0151 | 0.0296 | 6.09E-01 |
| Processed meat intake | rs11032380 | T | A | 35.920 | 7.89E-05 | -0.0133 | 0.0022 | 2.10E-09 | 0.0003 | 0.0189 | 9.87E-01 |
| Processed meat intake | rs11887120 | T | C | 30.657 | 6.85E-05 | 0.0120 | 0.0022 | 3.10E-08 | 0.0403 | 0.0185 | 2.97E-02 |
| Processed meat intake | rs11894162 | T | C | 32.702 | 7.19E-05 | 0.0120 | 0.0021 | 1.10E-08 | -0.0026 | 0.0182 | 8.85E-01 |
| Processed meat intake | rs1422192 | A | G | 34.923 | 7.66E-05 | 0.0170 | 0.0029 | 3.40E-09 | 0.0005 | 0.0242 | 9.85E-01 |
| Processed meat intake | rs2029401 | G | A | 47.220 | 1.04E-04 | 0.0146 | 0.0021 | 6.30E-12 | -0.0099 | 0.0191 | 6.03E-01 |
| Processed meat intake | rs203319 | T | C | 39.842 | 8.78E-05 | -0.0164 | 0.0026 | 2.80E-10 | -0.0273 | 0.0223 | 2.20E-01 |
| Processed meat intake | rs2873054 | C | A | 40.847 | 8.95E-05 | 0.0140 | 0.0022 | 1.60E-10 | 0.0040 | 0.0191 | 8.35E-01 |
| Processed meat intake | rs34241936 | G | A | 32.574 | 7.76E-05 | 0.0328 | 0.0058 | 1.10E-08 | 0.0245 | 0.0558 | 6.60E-01 |
| Processed meat intake | rs3762621 | T | C | 30.377 | 6.73E-05 | -0.0150 | 0.0027 | 3.60E-08 | 0.0112 | 0.0234 | 6.32E-01 |
| Processed meat intake | rs4077924 | C | T | 29.928 | 6.54E-05 | 0.0125 | 0.0023 | 4.50E-08 | 0.0100 | 0.0199 | 6.15E-01 |
| Processed meat intake | rs4240672 | A | G | 66.836 | 1.47E-04 | 0.0171 | 0.0021 | 3.00E-16 | 0.0321 | 0.0180 | 7.51E-02 |
| Processed meat intake | rs4778053 | G | C | 32.270 | 7.15E-05 | 0.0165 | 0.0029 | 1.30E-08 | 0.0176 | 0.0253 | 4.85E-01 |
| Processed meat intake | rs6010651 | C | A | 32.746 | 7.25E-05 | -0.0124 | 0.0022 | 1.10E-08 | 0.0081 | 0.0186 | 6.63E-01 |
| Processed meat intake | rs6484504 | C | T | 43.429 | 9.55E-05 | 0.0155 | 0.0023 | 4.40E-11 | 0.0067 | 0.0206 | 7.47E-01 |
| Processed meat intake | rs6765179 | A | G | 31.709 | 6.97E-05 | -0.0128 | 0.0023 | 1.80E-08 | -0.0036 | 0.0199 | 8.54E-01 |
| Processed meat intake | rs6786550 | C | T | 31.423 | 6.88E-05 | 0.0122 | 0.0022 | 2.10E-08 | 0.0013 | 0.0190 | 9.45E-01 |
| Processed meat intake | rs6961970 | A | C | 32.951 | 7.26E-05 | -0.0140 | 0.0024 | 9.50E-09 | -0.0453 | 0.0221 | 4.01E-02 |
| Processed meat intake | rs7531118 | C | T | 44.332 | 9.86E-05 | -0.0141 | 0.0021 | 2.80E-11 | -0.0030 | 0.0185 | 8.69E-01 |
| Processed meat intake | rs77165542 | T | C | 35.018 | 7.86E-05 | 0.0339 | 0.0057 | 3.30E-09 | -0.0303 | 0.0567 | 5.93E-01 |
| Processed meat intake | rs8096167 | C | T | 29.856 | 6.62E-05 | -0.0146 | 0.0027 | 4.70E-08 | 0.0129 | 0.0232 | 5.78E-01 |
| Processed meat intake | rs838133 | G | A | 77.090 | 1.79E-04 | 0.0190 | 0.0022 | 1.60E-18 | -0.0312 | 0.0200 | 1.19E-01 |
| Processed meat intake | rs9809856 | G | A | 40.056 | 8.81E-05 | 0.0133 | 0.0021 | 2.50E-10 | 0.0538 | 0.0182 | 3.07E-03 |
| Poultry intake | rs1051730 | A | G | 31.776 | 5.24E-05 | -0.0109 | 0.0019 | 1.70E-08 | 0.0008 | 0.0189 | 9.68E-01 |
| Poultry intake | rs2426440 | G | A | 29.849 | 4.93E-05 | 0.0112 | 0.0021 | 4.70E-08 | 0.0382 | 0.0205 | 6.19E-02 |
| Poultry intake | rs2565017 | A | G | 33.858 | 5.61E-05 | 0.0110 | 0.0019 | 5.90E-09 | -0.0169 | 0.0188 | 3.68E-01 |
| Poultry intake | rs2965200 | A | G | 30.047 | 5.00E-05 | -0.0104 | 0.0019 | 4.20E-08 | 0.0112 | 0.0191 | 5.56E-01 |
| Poultry intake | rs7829800 | G | A | 34.781 | 5.80E-05 | 0.0115 | 0.0019 | 3.70E-09 | 0.0303 | 0.0191 | 1.12E-01 |
| Poultry intake | rs9923768 | A | G | 31.977 | 5.31E-05 | 0.0105 | 0.0019 | 1.60E-08 | 0.0085 | 0.0185 | 6.48E-01 |
| Poultry intake | rs9997448 | T | C | 30.889 | 5.10E-05 | -0.0105 | 0.0019 | 2.70E-08 | -0.0048 | 0.0191 | 8.01E-01 |
| Beef intake | rs10789340 | G | A | 60.652 | 8.88E-05 | -0.0138 | 0.0018 | 6.80E-15 | -0.0337 | 0.0191 | 7.74E-02 |
| Beef intake | rs10959890 | C | T | 36.512 | 5.37E-05 | -0.0127 | 0.0021 | 1.50E-09 | -0.0280 | 0.0224 | 2.10E-01 |
| Beef intake | rs1105388 | T | C | 36.782 | 5.44E-05 | -0.0114 | 0.0019 | 1.30E-09 | -0.0015 | 0.0200 | 9.39E-01 |
| Beef intake | rs11165829 | G | C | 32.887 | 4.79E-05 | -0.0102 | 0.0018 | 9.80E-09 | -0.0280 | 0.0186 | 1.33E-01 |
| Beef intake | rs11878917 | A | G | 29.867 | 4.41E-05 | 0.0150 | 0.0027 | 4.60E-08 | -0.0082 | 0.0291 | 7.79E-01 |
| Beef intake | rs132901 | T | C | 44.271 | 6.49E-05 | 0.0139 | 0.0021 | 2.90E-11 | 0.0222 | 0.0219 | 3.10E-01 |
| Beef intake | rs1421085 | C | T | 48.392 | 7.07E-05 | -0.0121 | 0.0017 | 3.50E-12 | -0.0017 | 0.0184 | 9.27E-01 |
| Beef intake | rs1470610 | C | G | 32.050 | 4.71E-05 | -0.0122 | 0.0022 | 1.50E-08 | -0.0155 | 0.0224 | 4.89E-01 |
| Beef intake | rs429358 | C | T | 39.319 | 5.75E-05 | -0.0149 | 0.0024 | 3.60E-10 | -0.0008 | 0.0267 | 9.75E-01 |
| Beef intake | rs4676964 | T | C | 59.967 | 8.91E-05 | 0.0134 | 0.0017 | 9.60E-15 | -0.0177 | 0.0183 | 3.33E-01 |
| Beef intake | rs62169335 | T | C | 31.160 | 4.66E-05 | -0.0097 | 0.0017 | 2.40E-08 | -0.0097 | 0.0187 | 6.04E-01 |
| Beef intake | rs62396185 | C | G | 57.892 | 8.48E-05 | -0.0148 | 0.0020 | 2.80E-14 | -0.0182 | 0.0202 | 3.68E-01 |
| Beef intake | rs7791463 | A | G | 31.157 | 4.54E-05 | 0.0095 | 0.0017 | 2.40E-08 | -0.0128 | 0.0180 | 4.75E-01 |
| Beef intake | rs784251 | T | C | 36.278 | 5.33E-05 | -0.0103 | 0.0017 | 1.70E-09 | 0.0004 | 0.0180 | 9.84E-01 |
| Beef intake | rs79809011 | A | G | 30.451 | 4.50E-05 | -0.0281 | 0.0051 | 3.40E-08 | -0.0211 | 0.0623 | 7.35E-01 |
| Non-oily fish intake | rs11680516 | C | T | 36.680 | 4.87E-05 | 0.0123 | 0.0020 | 1.40E-09 | -0.0194 | 0.0231 | 4.01E-01 |
| Non-oily fish intake | rs1260326 | C | T | 33.310 | 4.37E-05 | -0.0096 | 0.0017 | 7.90E-09 | 0.0557 | 0.0182 | 2.27E-03 |
| Non-oily fish intake | rs16822430 | C | T | 36.720 | 4.84E-05 | 0.0116 | 0.0019 | 1.40E-09 | -0.0146 | 0.0212 | 4.90E-01 |
| Non-oily fish intake | rs17317920 | G | A | 30.851 | 4.10E-05 | 0.0091 | 0.0016 | 2.80E-08 | 0.0262 | 0.0184 | 1.55E-01 |
| Non-oily fish intake | rs35287743 | T | G | 48.306 | 6.45E-05 | -0.0177 | 0.0026 | 3.60E-12 | 0.0210 | 0.0283 | 4.58E-01 |
| Non-oily fish intake | rs3799077 | G | T | 37.300 | 4.93E-05 | -0.0107 | 0.0018 | 1.00E-09 | -0.0051 | 0.0194 | 7.93E-01 |
| Non-oily fish intake | rs4318925 | T | C | 50.281 | 6.60E-05 | -0.0150 | 0.0021 | 1.30E-12 | 0.0335 | 0.0245 | 1.72E-01 |
| Non-oily fish intake | rs56094641 | G | A | 58.122 | 7.63E-05 | 0.0126 | 0.0017 | 2.50E-14 | -0.0026 | 0.0183 | 8.88E-01 |
| Non-oily fish intake | rs6957745 | C | T | 36.233 | 4.80E-05 | -0.0122 | 0.0020 | 1.80E-09 | 0.0147 | 0.0233 | 5.27E-01 |
| Non-oily fish intake | rs7148387 | G | A | 31.804 | 4.19E-05 | -0.0093 | 0.0017 | 1.70E-08 | 0.0081 | 0.0191 | 6.71E-01 |
| Non-oily fish intake | rs838133 | G | A | 93.217 | 1.30E-04 | 0.0162 | 0.0017 | 4.70E-22 | -0.0312 | 0.0200 | 1.19E-01 |
| Oily fish intake | rs10061973 | T | G | 32.081 | 5.88E-05 | -0.0109 | 0.0019 | 1.50E-08 | 0.0029 | 0.0182 | 8.75E-01 |
| Oily fish intake | rs10076975 | C | T | 32.643 | 5.96E-05 | 0.0112 | 0.0020 | 1.10E-08 | -0.0031 | 0.0185 | 8.67E-01 |
| Oily fish intake | rs10510554 | C | T | 32.533 | 5.98E-05 | 0.0110 | 0.0019 | 1.20E-08 | 0.0108 | 0.0182 | 5.55E-01 |
| Oily fish intake | rs10513136 | A | G | 36.451 | 6.65E-05 | -0.0233 | 0.0039 | 1.60E-09 | 0.0227 | 0.0417 | 5.85E-01 |
| Oily fish intake | rs10828250 | G | C | 94.410 | 1.73E-04 | -0.0201 | 0.0021 | 2.60E-22 | 0.0264 | 0.0209 | 2.06E-01 |
| Oily fish intake | rs114497213 | T | G | 41.602 | 7.73E-05 | 0.0273 | 0.0042 | 1.10E-10 | 0.0050 | 0.0402 | 9.00E-01 |
| Oily fish intake | rs11767283 | G | A | 58.061 | 1.08E-04 | 0.0177 | 0.0023 | 2.50E-14 | -0.0055 | 0.0230 | 8.09E-01 |
| Oily fish intake | rs11859365 | C | A | 105.528 | 1.93E-04 | 0.0226 | 0.0022 | 9.40E-25 | 0.0178 | 0.0211 | 3.98E-01 |
| Oily fish intake | rs1201289 | G | T | 29.966 | 5.50E-05 | -0.0107 | 0.0020 | 4.40E-08 | 0.0160 | 0.0187 | 3.93E-01 |
| Oily fish intake | rs12663865 | A | G | 32.732 | 5.98E-05 | 0.0128 | 0.0022 | 1.10E-08 | -0.0305 | 0.0208 | 1.42E-01 |
| Oily fish intake | rs12855717 | T | C | 40.457 | 7.46E-05 | -0.0122 | 0.0019 | 2.00E-10 | -0.0040 | 0.0181 | 8.26E-01 |
| Oily fish intake | rs12896749 | C | G | 31.099 | 5.69E-05 | -0.0110 | 0.0020 | 2.50E-08 | 0.0261 | 0.0187 | 1.62E-01 |
| Oily fish intake | rs12983532 | T | C | 35.944 | 6.74E-05 | -0.0134 | 0.0022 | 2.00E-09 | -0.0079 | 0.0222 | 7.21E-01 |
| Oily fish intake | rs13070166 | A | T | 38.911 | 7.12E-05 | 0.0142 | 0.0023 | 4.40E-10 | -0.0272 | 0.0212 | 2.00E-01 |
| Oily fish intake | rs1361016 | G | T | 31.784 | 5.86E-05 | 0.0150 | 0.0027 | 1.70E-08 | -0.0222 | 0.0248 | 3.71E-01 |
| Oily fish intake | rs1421085 | C | T | 89.943 | 1.64E-04 | 0.0185 | 0.0019 | 2.50E-21 | -0.0017 | 0.0184 | 9.27E-01 |
| Oily fish intake | rs16891727 | A | C | 69.740 | 1.27E-04 | -0.0237 | 0.0028 | 6.80E-17 | 0.0193 | 0.0294 | 5.12E-01 |
| Oily fish intake | rs17050031 | T | C | 39.355 | 7.25E-05 | -0.0120 | 0.0019 | 3.50E-10 | 0.0075 | 0.0180 | 6.77E-01 |
| Oily fish intake | rs1876245 | C | T | 61.266 | 1.12E-04 | 0.0151 | 0.0019 | 5.00E-15 | 0.0342 | 0.0182 | 6.01E-02 |
| Oily fish intake | rs1951286 | G | T | 53.196 | 9.74E-05 | -0.0146 | 0.0020 | 3.00E-13 | 0.0213 | 0.0195 | 2.76E-01 |
| Oily fish intake | rs2374424 | G | A | 34.226 | 6.28E-05 | -0.0114 | 0.0020 | 4.90E-09 | 0.0143 | 0.0186 | 4.40E-01 |
| Oily fish intake | rs275160 | C | T | 33.272 | 6.16E-05 | 0.0121 | 0.0021 | 8.00E-09 | -0.0081 | 0.0198 | 6.82E-01 |
| Oily fish intake | rs2827161 | G | T | 30.574 | 5.60E-05 | 0.0107 | 0.0019 | 3.20E-08 | -0.0098 | 0.0183 | 5.92E-01 |
| Oily fish intake | rs28533540 | A | G | 57.869 | 1.07E-04 | 0.0146 | 0.0019 | 2.80E-14 | 0.0042 | 0.0181 | 8.15E-01 |
| Oily fish intake | rs28623270 | T | A | 42.437 | 8.05E-05 | -0.0178 | 0.0027 | 7.30E-11 | 0.0648 | 0.0270 | 1.65E-02 |
| Oily fish intake | rs303817 | G | A | 37.770 | 6.89E-05 | 0.0136 | 0.0022 | 8.00E-10 | 0.0050 | 0.0209 | 8.11E-01 |
| Oily fish intake | rs3124402 | G | A | 104.087 | 1.89E-04 | -0.0220 | 0.0022 | 1.90E-24 | 0.0265 | 0.0205 | 1.97E-01 |
| Oily fish intake | rs35287743 | T | G | 87.858 | 1.63E-04 | -0.0282 | 0.0030 | 7.00E-21 | 0.0210 | 0.0283 | 4.58E-01 |
| Oily fish intake | rs4002471 | T | C | 99.999 | 1.84E-04 | -0.0192 | 0.0019 | 1.50E-23 | 0.0260 | 0.0188 | 1.66E-01 |
| Oily fish intake | rs4278546 | G | A | 41.969 | 7.77E-05 | 0.0126 | 0.0019 | 9.30E-11 | -0.0083 | 0.0182 | 6.50E-01 |
| Oily fish intake | rs4510068 | T | G | 43.601 | 8.15E-05 | -0.0130 | 0.0020 | 4.00E-11 | -0.0347 | 0.0183 | 5.85E-02 |
| Oily fish intake | rs45501495 | T | C | 48.261 | 8.87E-05 | 0.0157 | 0.0023 | 3.70E-12 | -0.0254 | 0.0212 | 2.31E-01 |
| Oily fish intake | rs4869859 | C | T | 53.128 | 9.72E-05 | 0.0140 | 0.0019 | 3.10E-13 | -0.0070 | 0.0180 | 6.98E-01 |
| Oily fish intake | rs4982738 | A | G | 30.414 | 5.74E-05 | 0.0109 | 0.0020 | 3.50E-08 | 0.0241 | 0.0199 | 2.26E-01 |
| Oily fish intake | rs510161 | G | C | 29.910 | 5.47E-05 | -0.0113 | 0.0021 | 4.50E-08 | -0.0162 | 0.0194 | 4.05E-01 |
| Oily fish intake | rs552234 | A | G | 37.089 | 6.78E-05 | -0.0116 | 0.0019 | 1.10E-09 | 0.0259 | 0.0181 | 1.52E-01 |
| Oily fish intake | rs55930451 | T | C | 30.745 | 5.60E-05 | -0.0171 | 0.0031 | 2.90E-08 | 0.0548 | 0.0297 | 6.49E-02 |
| Oily fish intake | rs55985303 | A | G | 33.657 | 6.16E-05 | 0.0130 | 0.0022 | 6.60E-09 | 0.0631 | 0.0211 | 2.80E-03 |
| Oily fish intake | rs59355765 | T | C | 38.784 | 7.10E-05 | -0.0163 | 0.0026 | 4.70E-10 | 0.0098 | 0.0248 | 6.93E-01 |
| Oily fish intake | rs6033437 | A | C | 31.836 | 5.94E-05 | 0.0125 | 0.0022 | 1.70E-08 | 0.0066 | 0.0206 | 7.47E-01 |
| Oily fish intake | rs6059844 | G | A | 33.012 | 6.05E-05 | 0.0110 | 0.0019 | 9.20E-09 | -0.0390 | 0.0182 | 3.21E-02 |
| Oily fish intake | rs6089753 | T | C | 36.198 | 6.64E-05 | -0.0115 | 0.0019 | 1.80E-09 | -0.0065 | 0.0189 | 7.32E-01 |
| Oily fish intake | rs61882686 | A | C | 33.264 | 6.09E-05 | 0.0198 | 0.0034 | 8.00E-09 | -0.0343 | 0.0327 | 2.94E-01 |
| Oily fish intake | rs631490 | C | G | 51.851 | 9.46E-05 | -0.0151 | 0.0021 | 6.00E-13 | -0.0188 | 0.0194 | 3.34E-01 |
| Oily fish intake | rs6465487 | G | A | 39.877 | 7.32E-05 | -0.0124 | 0.0020 | 2.70E-10 | 0.0313 | 0.0184 | 8.96E-02 |
| Oily fish intake | rs703987 | C | G | 31.756 | 5.86E-05 | 0.0111 | 0.0020 | 1.70E-08 | -0.0084 | 0.0188 | 6.56E-01 |
| Oily fish intake | rs7243428 | G | A | 32.060 | 5.87E-05 | -0.0130 | 0.0023 | 1.50E-08 | 0.0056 | 0.0217 | 7.95E-01 |
| Oily fish intake | rs7254235 | G | A | 30.022 | 5.52E-05 | -0.0106 | 0.0019 | 4.30E-08 | -0.0142 | 0.0182 | 4.33E-01 |
| Oily fish intake | rs75887709 | G | A | 31.951 | 5.94E-05 | -0.0159 | 0.0028 | 1.60E-08 | 0.0171 | 0.0296 | 5.64E-01 |
| Oily fish intake | rs7683782 | G | C | 31.635 | 5.82E-05 | 0.0145 | 0.0026 | 1.90E-08 | -0.0294 | 0.0246 | 2.32E-01 |
| Oily fish intake | rs790564 | C | A | 46.789 | 8.65E-05 | 0.0147 | 0.0021 | 7.90E-12 | 0.0285 | 0.0202 | 1.58E-01 |
| Oily fish intake | rs905575 | G | C | 30.368 | 5.59E-05 | 0.0139 | 0.0025 | 3.60E-08 | 0.0073 | 0.0236 | 7.56E-01 |
| Oily fish intake | rs9301837 | A | C | 33.243 | 6.08E-05 | -0.0157 | 0.0027 | 8.10E-09 | -0.0054 | 0.0256 | 8.34E-01 |
| Oily fish intake | rs9597870 | G | T | 32.650 | 6.02E-05 | -0.0127 | 0.0022 | 1.10E-08 | -0.0056 | 0.0214 | 7.93E-01 |
| Oily fish intake | rs9606833 | C | T | 57.971 | 1.06E-04 | 0.0170 | 0.0022 | 2.70E-14 | -0.0016 | 0.0214 | 9.41E-01 |
| Oily fish intake | rs973526 | T | C | 35.562 | 6.62E-05 | -0.0115 | 0.0019 | 2.50E-09 | -0.0166 | 0.0184 | 3.68E-01 |
| Oily fish intake | rs9841174 | C | T | 55.693 | 1.02E-04 | 0.0148 | 0.0020 | 8.50E-14 | -0.0160 | 0.0187 | 3.92E-01 |
| Oily fish intake | rs9889161 | T | G | 44.290 | 8.16E-05 | -0.0133 | 0.0020 | 2.80E-11 | 0.0072 | 0.0189 | 7.02E-01 |
| Oily fish intake | rs9958909 | G | T | 32.189 | 5.97E-05 | 0.0158 | 0.0028 | 1.40E-08 | 0.0276 | 0.0259 | 2.86E-01 |
| Pork intake | rs10972033 | T | G | 36.853 | 4.00E-05 | 0.0090 | 0.0015 | 1.30E-09 | -0.0038 | 0.0180 | 8.34E-01 |
| Pork intake | rs11211124 | C | T | 32.195 | 3.52E-05 | -0.0100 | 0.0018 | 1.40E-08 | -0.0105 | 0.0215 | 6.25E-01 |
| Pork intake | rs12721051 | G | C | 42.945 | 4.67E-05 | -0.0124 | 0.0019 | 5.60E-11 | -0.0009 | 0.0261 | 9.73E-01 |
| Pork intake | rs1355171 | A | C | 55.296 | 6.03E-05 | -0.0110 | 0.0015 | 1.00E-13 | 0.0066 | 0.0180 | 7.13E-01 |
| Pork intake | rs2387807 | T | C | 30.124 | 3.27E-05 | -0.0151 | 0.0027 | 4.10E-08 | -0.0218 | 0.0318 | 4.93E-01 |
| Pork intake | rs254152 | G | C | 35.818 | 3.91E-05 | -0.0104 | 0.0017 | 2.20E-09 | 0.0377 | 0.0213 | 7.65E-02 |
| Pork intake | rs34161520 | G | C | 32.912 | 3.62E-05 | 0.0116 | 0.0020 | 9.60E-09 | 0.0271 | 0.0243 | 2.65E-01 |
| Pork intake | rs36124222 | C | T | 31.395 | 3.47E-05 | 0.0084 | 0.0015 | 2.10E-08 | 0.0089 | 0.0205 | 6.63E-01 |
| Pork intake | rs3964074 | C | T | 36.406 | 3.97E-05 | -0.0090 | 0.0015 | 1.60E-09 | -0.0013 | 0.0183 | 9.44E-01 |
| Pork intake | rs4146837 | T | C | 34.615 | 3.83E-05 | 0.0088 | 0.0015 | 4.00E-09 | 0.0215 | 0.0181 | 2.34E-01 |
| Pork intake | rs7641973 | A | G | 30.042 | 3.26E-05 | 0.0084 | 0.0015 | 4.20E-08 | 0.0048 | 0.0191 | 8.01E-01 |
| Pork intake | rs838133 | G | A | 51.041 | 5.88E-05 | 0.0109 | 0.0015 | 9.00E-13 | -0.0312 | 0.0200 | 1.19E-01 |
| Pork intake | rs9379832 | G | A | 45.200 | 5.01E-05 | -0.0115 | 0.0017 | 1.80E-11 | -0.0206 | 0.0204 | 3.14E-01 |
| Pork intake | rs9973426 | G | A | 32.756 | 3.57E-05 | 0.0111 | 0.0019 | 1.00E-08 | 0.0061 | 0.0234 | 7.93E-01 |
| Lamb/mutton intake | rs11090045 | A | G | 44.160 | 4.85E-05 | -0.0107 | 0.0016 | 3.00E-11 | 0.0113 | 0.0202 | 5.77E-01 |
| Lamb/mutton intake | rs11743441 | T | G | 35.408 | 3.82E-05 | -0.0088 | 0.0015 | 2.70E-09 | -0.0114 | 0.0183 | 5.33E-01 |
| Lamb/mutton intake | rs12634740 | G | T | 35.324 | 3.84E-05 | -0.0101 | 0.0017 | 2.80E-09 | -0.0109 | 0.0211 | 6.06E-01 |
| Lamb/mutton intake | rs136548 | T | C | 39.733 | 4.27E-05 | 0.0095 | 0.0015 | 2.90E-10 | -0.0028 | 0.0186 | 8.81E-01 |
| Lamb/mutton intake | rs139237013 | A | G | 36.224 | 3.88E-05 | 0.0189 | 0.0031 | 1.80E-09 | 0.0030 | 0.0437 | 9.45E-01 |
| Lamb/mutton intake | rs1556147 | T | A | 34.078 | 3.65E-05 | 0.0091 | 0.0016 | 5.30E-09 | -0.0187 | 0.0194 | 3.35E-01 |
| Lamb/mutton intake | rs16891982 | G | C | 30.919 | 3.19E-05 | -0.0243 | 0.0044 | 2.70E-08 | -0.0047 | 0.0513 | 9.27E-01 |
| Lamb/mutton intake | rs17270057 | C | T | 30.031 | 3.22E-05 | 0.0127 | 0.0023 | 4.30E-08 | 0.0014 | 0.0287 | 9.61E-01 |
| Lamb/mutton intake | rs1958801 | G | A | 30.575 | 3.28E-05 | -0.0089 | 0.0016 | 3.20E-08 | -0.0094 | 0.0205 | 6.47E-01 |
| Lamb/mutton intake | rs2222760 | A | G | 30.844 | 3.34E-05 | -0.0091 | 0.0016 | 2.80E-08 | 0.0152 | 0.0202 | 4.53E-01 |
| Lamb/mutton intake | rs2678900 | G | T | 46.353 | 4.98E-05 | 0.0101 | 0.0015 | 9.90E-12 | -0.0072 | 0.0183 | 6.94E-01 |
| Lamb/mutton intake | rs2726033 | G | A | 41.091 | 4.39E-05 | -0.0095 | 0.0015 | 1.50E-10 | -0.0010 | 0.0190 | 9.57E-01 |
| Lamb/mutton intake | rs276453 | C | A | 94.154 | 1.01E-04 | -0.0142 | 0.0015 | 2.90E-22 | 0.0081 | 0.0180 | 6.52E-01 |
| Lamb/mutton intake | rs2926119 | A | C | 29.964 | 3.23E-05 | 0.0081 | 0.0015 | 4.40E-08 | -0.0085 | 0.0183 | 6.43E-01 |
| Lamb/mutton intake | rs3105056 | C | T | 49.719 | 5.29E-05 | -0.0116 | 0.0016 | 1.80E-12 | 0.0270 | 0.0205 | 1.88E-01 |
| Lamb/mutton intake | rs35797675 | G | T | 36.601 | 3.99E-05 | -0.0108 | 0.0018 | 1.40E-09 | 0.0215 | 0.0229 | 3.46E-01 |
| Lamb/mutton intake | rs3964074 | C | T | 30.581 | 3.28E-05 | -0.0081 | 0.0015 | 3.20E-08 | -0.0013 | 0.0183 | 9.44E-01 |
| Lamb/mutton intake | rs4272399 | A | C | 34.406 | 3.73E-05 | -0.0092 | 0.0016 | 4.50E-09 | 0.0304 | 0.0192 | 1.13E-01 |
| Lamb/mutton intake | rs429358 | C | T | 80.612 | 8.64E-05 | -0.0182 | 0.0020 | 2.70E-19 | -0.0008 | 0.0267 | 9.75E-01 |
| Lamb/mutton intake | rs4489752 | T | G | 48.858 | 5.23E-05 | 0.0138 | 0.0020 | 2.80E-12 | -0.0017 | 0.0246 | 9.44E-01 |
| Lamb/mutton intake | rs55813438 | A | G | 43.300 | 4.71E-05 | -0.0114 | 0.0017 | 4.70E-11 | 0.0397 | 0.0212 | 6.16E-02 |
| Lamb/mutton intake | rs56394517 | G | A | 30.574 | 3.29E-05 | -0.0138 | 0.0025 | 3.20E-08 | -0.0017 | 0.0298 | 9.53E-01 |
| Lamb/mutton intake | rs62106258 | C | T | 40.437 | 4.32E-05 | 0.0216 | 0.0034 | 2.00E-10 | -0.0041 | 0.0478 | 9.31E-01 |
| Lamb/mutton intake | rs62398404 | T | C | 34.642 | 3.70E-05 | 0.0129 | 0.0022 | 4.00E-09 | 0.0090 | 0.0279 | 7.45E-01 |
| Lamb/mutton intake | rs6581296 | G | C | 30.166 | 3.28E-05 | 0.0100 | 0.0018 | 4.00E-08 | 0.0319 | 0.0224 | 1.54E-01 |
| Lamb/mutton intake | rs660880 | A | G | 38.064 | 4.08E-05 | -0.0090 | 0.0015 | 6.80E-10 | 0.0314 | 0.0179 | 7.97E-02 |
| Lamb/mutton intake | rs673696 | T | C | 34.796 | 3.73E-05 | 0.0158 | 0.0027 | 3.70E-09 | 0.0173 | 0.0325 | 5.95E-01 |
| Lamb/mutton intake | rs6829572 | A | G | 32.481 | 3.50E-05 | 0.0084 | 0.0015 | 1.20E-08 | -0.0060 | 0.0181 | 7.40E-01 |
| Lamb/mutton intake | rs7447465 | C | T | 40.474 | 4.33E-05 | 0.0096 | 0.0015 | 2.00E-10 | -0.0150 | 0.0185 | 4.15E-01 |
| Lamb/mutton intake | rs7550173 | T | A | 36.885 | 3.96E-05 | -0.0091 | 0.0015 | 1.30E-09 | -0.0179 | 0.0185 | 3.33E-01 |
| Lamb/mutton intake | rs994270 | G | C | 32.242 | 3.47E-05 | 0.0098 | 0.0017 | 1.40E-08 | -0.0262 | 0.0209 | 2.11E-01 |
| Bread intake | rs10761661 | T | C | 32.818 | 6.54E-05 | -0.0115 | 0.0020 | 1.00E-08 | -0.0128 | 0.0181 | 4.80E-01 |
| Bread intake | rs11060853 | G | A | 40.049 | 7.92E-05 | -0.0128 | 0.0020 | 2.50E-10 | -0.0002 | 0.0185 | 9.92E-01 |
| Bread intake | rs11183201 | C | T | 70.230 | 1.40E-04 | -0.0167 | 0.0020 | 5.30E-17 | 0.0007 | 0.0182 | 9.68E-01 |
| Bread intake | rs11628639 | C | T | 33.769 | 6.71E-05 | -0.0135 | 0.0023 | 6.20E-09 | 0.0085 | 0.0210 | 6.84E-01 |
| Bread intake | rs13016665 | A | C | 52.916 | 1.06E-04 | 0.0148 | 0.0020 | 3.50E-13 | 0.0037 | 0.0185 | 8.44E-01 |
| Bread intake | rs13023099 | A | C | 32.155 | 6.44E-05 | -0.0115 | 0.0020 | 1.40E-08 | -0.0176 | 0.0186 | 3.43E-01 |
| Bread intake | rs1492988 | G | C | 32.168 | 6.40E-05 | 0.0115 | 0.0020 | 1.40E-08 | -0.0160 | 0.0188 | 3.95E-01 |
| Bread intake | rs17083079 | A | G | 41.415 | 8.19E-05 | 0.0301 | 0.0047 | 1.20E-10 | -0.1247 | 0.0547 | 2.26E-02 |
| Bread intake | rs1940033 | T | C | 29.832 | 5.93E-05 | -0.0111 | 0.0020 | 4.70E-08 | 0.0312 | 0.0183 | 8.78E-02 |
| Bread intake | rs1994315 | C | T | 61.549 | 1.22E-04 | -0.0168 | 0.0021 | 4.30E-15 | 0.0264 | 0.0197 | 1.79E-01 |
| Bread intake | rs2068650 | C | A | 48.601 | 9.68E-05 | -0.0139 | 0.0020 | 3.10E-12 | 0.0075 | 0.0187 | 6.88E-01 |
| Bread intake | rs2517678 | T | C | 40.244 | 8.15E-05 | 0.0132 | 0.0021 | 2.20E-10 | -0.0021 | 0.0189 | 9.12E-01 |
| Bread intake | rs28406095 | A | G | 29.967 | 5.96E-05 | -0.0109 | 0.0020 | 4.40E-08 | -0.0104 | 0.0181 | 5.66E-01 |
| Bread intake | rs4665972 | C | T | 48.540 | 9.69E-05 | -0.0142 | 0.0020 | 3.20E-12 | 0.0634 | 0.0189 | 7.68E-04 |
| Bread intake | rs4984685 | A | G | 29.986 | 5.93E-05 | 0.0136 | 0.0025 | 4.40E-08 | -0.0061 | 0.0237 | 7.96E-01 |
| Bread intake | rs55745436 | T | C | 32.839 | 6.52E-05 | 0.0134 | 0.0023 | 1.00E-08 | -0.0165 | 0.0215 | 4.44E-01 |
| Bread intake | rs596878 | C | A | 34.086 | 6.82E-05 | -0.0117 | 0.0020 | 5.30E-09 | -0.0115 | 0.0181 | 5.26E-01 |
| Bread intake | rs62091167 | C | A | 32.565 | 6.49E-05 | -0.0138 | 0.0024 | 1.20E-08 | -0.0253 | 0.0218 | 2.45E-01 |
| Bread intake | rs656817 | G | A | 36.185 | 7.17E-05 | -0.0127 | 0.0021 | 1.80E-09 | -0.0001 | 0.0193 | 9.95E-01 |
| Bread intake | rs6580721 | G | A | 45.754 | 9.06E-05 | 0.0172 | 0.0025 | 1.30E-11 | 0.0020 | 0.0257 | 9.37E-01 |
| Bread intake | rs6754311 | C | T | 39.589 | 7.77E-05 | 0.0141 | 0.0022 | 3.10E-10 | 0.0395 | 0.0206 | 5.52E-02 |
| Bread intake | rs73802707 | T | C | 33.272 | 6.60E-05 | -0.0159 | 0.0028 | 8.00E-09 | 0.0050 | 0.0252 | 8.41E-01 |
| Bread intake | rs7802468 | T | C | 128.975 | 2.55E-04 | -0.0234 | 0.0021 | 6.90E-30 | -0.0131 | 0.0191 | 4.94E-01 |
| Bread intake | rs79436018 | C | T | 31.947 | 6.38E-05 | -0.0176 | 0.0031 | 1.60E-08 | -0.0121 | 0.0283 | 6.69E-01 |
| Bread intake | rs9323989 | C | T | 31.927 | 6.35E-05 | -0.0116 | 0.0021 | 1.60E-08 | 0.0079 | 0.0184 | 6.67E-01 |
| Bread intake | rs9529024 | T | A | 39.017 | 7.78E-05 | -0.0129 | 0.0021 | 4.20E-10 | -0.0249 | 0.0187 | 1.82E-01 |
| Bread intake | rs9564268 | C | T | 35.212 | 7.00E-05 | -0.0122 | 0.0020 | 3.00E-09 | -0.0207 | 0.0189 | 2.75E-01 |
| Bread intake | rs9662365 | T | C | 37.406 | 7.40E-05 | 0.0122 | 0.0020 | 9.60E-10 | -0.0234 | 0.0181 | 1.97E-01 |
| Bread intake | rs9881332 | G | C | 31.625 | 6.29E-05 | 0.0114 | 0.0020 | 1.90E-08 | -0.0053 | 0.0186 | 7.75E-01 |
| Cheese intake | rs1073242 | A | G | 47.107 | 1.22E-04 | 0.0157 | 0.0023 | 6.70E-12 | 0.0122 | 0.0185 | 5.10E-01 |
| Cheese intake | rs10896050 | T | G | 42.461 | 1.06E-04 | -0.0185 | 0.0028 | 7.20E-11 | 0.0056 | 0.0233 | 8.11E-01 |
| Cheese intake | rs10938397 | G | A | 31.684 | 7.93E-05 | -0.0127 | 0.0023 | 1.80E-08 | 0.0288 | 0.0182 | 1.13E-01 |
| Cheese intake | rs113367286 | T | C | 36.872 | 9.26E-05 | 0.0152 | 0.0025 | 1.30E-09 | 0.0114 | 0.0223 | 6.09E-01 |
| Cheese intake | rs11620149 | C | T | 30.345 | 7.67E-05 | -0.0177 | 0.0032 | 3.60E-08 | 0.0016 | 0.0271 | 9.54E-01 |
| Cheese intake | rs11649653 | G | C | 36.517 | 9.06E-05 | 0.0138 | 0.0023 | 1.50E-09 | -0.0444 | 0.0188 | 1.79E-02 |
| Cheese intake | rs12296440 | A | G | 39.779 | 9.95E-05 | 0.0188 | 0.0030 | 2.80E-10 | -0.0122 | 0.0243 | 6.14E-01 |
| Cheese intake | rs12447542 | A | G | 33.596 | 8.56E-05 | 0.0197 | 0.0034 | 6.80E-09 | 0.0209 | 0.0287 | 4.66E-01 |
| Cheese intake | rs12475594 | G | A | 29.945 | 7.51E-05 | 0.0160 | 0.0029 | 4.40E-08 | 0.0236 | 0.0232 | 3.10E-01 |
| Cheese intake | rs12672200 | A | G | 33.037 | 8.32E-05 | -0.0138 | 0.0024 | 9.00E-09 | 0.0157 | 0.0205 | 4.43E-01 |
| Cheese intake | rs12786959 | T | A | 32.481 | 8.15E-05 | -0.0161 | 0.0028 | 1.20E-08 | 0.0018 | 0.0229 | 9.38E-01 |
| Cheese intake | rs1291145 | C | T | 70.583 | 1.77E-04 | -0.0202 | 0.0024 | 4.40E-17 | -0.0146 | 0.0194 | 4.53E-01 |
| Cheese intake | rs12951057 | G | C | 48.310 | 1.24E-04 | -0.0212 | 0.0030 | 3.60E-12 | -0.0004 | 0.0268 | 9.88E-01 |
| Cheese intake | rs13107325 | T | C | 47.025 | 1.18E-04 | -0.0292 | 0.0043 | 7.00E-12 | -0.0286 | 0.0352 | 4.17E-01 |
| Cheese intake | rs1434511 | T | C | 32.946 | 8.34E-05 | 0.0130 | 0.0023 | 9.50E-09 | 0.0071 | 0.0180 | 6.94E-01 |
| Cheese intake | rs1514755 | G | A | 39.166 | 9.77E-05 | 0.0164 | 0.0026 | 3.90E-10 | -0.0460 | 0.0212 | 2.99E-02 |
| Cheese intake | rs17115145 | T | C | 31.650 | 7.97E-05 | -0.0129 | 0.0023 | 1.80E-08 | 0.0150 | 0.0187 | 4.21E-01 |
| Cheese intake | rs1806771 | G | T | 30.125 | 7.86E-05 | -0.0221 | 0.0040 | 4.10E-08 | 0.0376 | 0.0331 | 2.56E-01 |
| Cheese intake | rs1931805 | C | T | 31.940 | 7.98E-05 | 0.0126 | 0.0022 | 1.60E-08 | -0.0125 | 0.0181 | 4.88E-01 |
| Cheese intake | rs2339928 | A | G | 36.929 | 9.20E-05 | 0.0149 | 0.0024 | 1.20E-09 | 0.0148 | 0.0202 | 4.64E-01 |
| Cheese intake | rs2352974 | T | C | 41.782 | 1.05E-04 | -0.0145 | 0.0022 | 1.00E-10 | 0.0154 | 0.0185 | 4.04E-01 |
| Cheese intake | rs2802530 | A | G | 30.065 | 7.51E-05 | 0.0186 | 0.0034 | 4.20E-08 | 0.0080 | 0.0280 | 7.76E-01 |
| Cheese intake | rs2854175 | A | C | 43.772 | 1.10E-04 | 0.0170 | 0.0026 | 3.70E-11 | 0.0218 | 0.0207 | 2.92E-01 |
| Cheese intake | rs2960578 | G | T | 57.999 | 1.45E-04 | 0.0170 | 0.0022 | 2.60E-14 | -0.0158 | 0.0180 | 3.79E-01 |
| Cheese intake | rs34198643 | T | C | 38.874 | 9.70E-05 | -0.0167 | 0.0027 | 4.50E-10 | -0.0016 | 0.0220 | 9.42E-01 |
| Cheese intake | rs35270670 | G | A | 36.538 | 9.15E-05 | 0.0164 | 0.0027 | 1.50E-09 | -0.0293 | 0.0213 | 1.69E-01 |
| Cheese intake | rs3911016 | G | T | 38.548 | 9.70E-05 | 0.0214 | 0.0034 | 5.30E-10 | -0.0355 | 0.0273 | 1.93E-01 |
| Cheese intake | rs4296548 | G | T | 32.410 | 8.07E-05 | 0.0130 | 0.0023 | 1.20E-08 | 0.0186 | 0.0184 | 3.13E-01 |
| Cheese intake | rs4503172 | T | C | 31.943 | 8.00E-05 | 0.0130 | 0.0023 | 1.60E-08 | 0.0349 | 0.0185 | 5.96E-02 |
| Cheese intake | rs4681981 | A | C | 30.790 | 7.70E-05 | -0.0124 | 0.0022 | 2.90E-08 | 0.0069 | 0.0181 | 7.05E-01 |
| Cheese intake | rs4692708 | C | A | 32.368 | 8.19E-05 | 0.0147 | 0.0026 | 1.30E-08 | -0.0194 | 0.0214 | 3.64E-01 |
| Cheese intake | rs4776970 | T | A | 43.854 | 1.09E-04 | 0.0154 | 0.0023 | 3.50E-11 | -0.0524 | 0.0187 | 4.99E-03 |
| Cheese intake | rs4860341 | C | T | 31.351 | 7.86E-05 | 0.0244 | 0.0044 | 2.20E-08 | 0.0170 | 0.0369 | 6.45E-01 |
| Cheese intake | rs504675 | T | C | 137.315 | 3.44E-04 | 0.0274 | 0.0023 | 1.00E-31 | -0.0008 | 0.0189 | 9.64E-01 |
| Cheese intake | rs524468 | G | A | 31.154 | 7.82E-05 | -0.0142 | 0.0026 | 2.40E-08 | -0.0315 | 0.0207 | 1.28E-01 |
| Cheese intake | rs531358 | T | C | 31.733 | 7.89E-05 | 0.0132 | 0.0023 | 1.80E-08 | -0.0286 | 0.0191 | 1.33E-01 |
| Cheese intake | rs6126641 | A | G | 30.498 | 7.81E-05 | 0.0132 | 0.0024 | 3.30E-08 | -0.0204 | 0.0195 | 2.95E-01 |
| Cheese intake | rs61734410 | T | C | 40.319 | 1.05E-04 | 0.0167 | 0.0026 | 2.20E-10 | 0.0276 | 0.0237 | 2.44E-01 |
| Cheese intake | rs61953351 | T | G | 31.994 | 7.99E-05 | 0.0146 | 0.0026 | 1.50E-08 | 0.0064 | 0.0206 | 7.56E-01 |
| Cheese intake | rs62034322 | A | G | 36.715 | 9.15E-05 | -0.0139 | 0.0023 | 1.40E-09 | -0.0012 | 0.0186 | 9.48E-01 |
| Cheese intake | rs62236533 | A | G | 46.105 | 1.19E-04 | 0.0248 | 0.0036 | 1.10E-11 | 0.0386 | 0.0337 | 2.53E-01 |
| Cheese intake | rs62245792 | A | T | 32.145 | 8.20E-05 | -0.0179 | 0.0032 | 1.40E-08 | -0.0238 | 0.0261 | 3.62E-01 |
| Cheese intake | rs6685323 | T | C | 29.801 | 7.43E-05 | -0.0132 | 0.0024 | 4.80E-08 | 0.0172 | 0.0196 | 3.81E-01 |
| Cheese intake | rs67238148 | T | G | 37.146 | 9.31E-05 | 0.0165 | 0.0027 | 1.10E-09 | 0.0107 | 0.0220 | 6.27E-01 |
| Cheese intake | rs6774906 | C | A | 31.090 | 7.80E-05 | 0.0316 | 0.0057 | 2.50E-08 | -0.0390 | 0.0450 | 3.86E-01 |
| Cheese intake | rs6873324 | C | A | 30.190 | 7.61E-05 | -0.0125 | 0.0023 | 3.90E-08 | 0.0067 | 0.0183 | 7.14E-01 |
| Cheese intake | rs7012814 | A | G | 67.490 | 1.71E-04 | -0.0185 | 0.0023 | 2.10E-16 | 0.0044 | 0.0184 | 8.13E-01 |
| Cheese intake | rs71386942 | A | C | 32.851 | 8.22E-05 | 0.0145 | 0.0025 | 9.90E-09 | -0.0320 | 0.0203 | 1.15E-01 |
| Cheese intake | rs72970243 | A | G | 42.596 | 1.04E-04 | 0.0222 | 0.0034 | 6.70E-11 | 0.0183 | 0.0235 | 4.36E-01 |
| Cheese intake | rs7298331 | C | A | 32.646 | 8.30E-05 | -0.0132 | 0.0023 | 1.10E-08 | 0.0075 | 0.0190 | 6.94E-01 |
| Cheese intake | rs73024305 | C | G | 43.608 | 1.10E-04 | 0.0325 | 0.0049 | 4.00E-11 | 0.0604 | 0.0392 | 1.24E-01 |
| Cheese intake | rs73096946 | C | T | 45.086 | 1.12E-04 | -0.0206 | 0.0031 | 1.90E-11 | 0.0021 | 0.0248 | 9.34E-01 |
| Cheese intake | rs73335955 | C | T | 31.123 | 7.79E-05 | 0.0278 | 0.0050 | 2.40E-08 | -0.0181 | 0.0388 | 6.41E-01 |
| Cheese intake | rs7386207 | T | C | 30.330 | 7.68E-05 | -0.0125 | 0.0023 | 3.60E-08 | 0.0309 | 0.0181 | 8.87E-02 |
| Cheese intake | rs77742462 | G | A | 32.879 | 9.06E-05 | -0.0475 | 0.0083 | 9.80E-09 | -0.0677 | 0.0703 | 3.36E-01 |
| Cheese intake | rs78876700 | A | G | 30.467 | 7.76E-05 | 0.0181 | 0.0033 | 3.40E-08 | -0.0009 | 0.0273 | 9.74E-01 |
| Cheese intake | rs79184944 | A | T | 35.615 | 8.94E-05 | 0.0196 | 0.0033 | 2.40E-09 | 0.0424 | 0.0268 | 1.13E-01 |
| Cheese intake | rs7936836 | A | C | 48.962 | 1.23E-04 | 0.0159 | 0.0023 | 2.60E-12 | -0.0180 | 0.0184 | 3.28E-01 |
| Cheese intake | rs919109 | C | G | 37.792 | 9.50E-05 | 0.0199 | 0.0032 | 7.90E-10 | -0.0087 | 0.0258 | 7.35E-01 |
| Cheese intake | rs9504123 | C | A | 32.030 | 8.00E-05 | 0.0142 | 0.0025 | 1.50E-08 | 0.0224 | 0.0199 | 2.61E-01 |
| Cheese intake | rs9649582 | T | A | 36.737 | 9.26E-05 | -0.0146 | 0.0024 | 1.40E-09 | 0.0382 | 0.0197 | 5.28E-02 |
| Cheese intake | rs975303 | G | A | 53.564 | 1.34E-04 | 0.0213 | 0.0029 | 2.50E-13 | 0.0542 | 0.0238 | 2.26E-02 |
| Cooked vegetable intake | rs10156602 | G | A | 45.212 | 5.59E-05 | 0.0110 | 0.0016 | 1.80E-11 | -0.0118 | 0.0187 | 5.30E-01 |
| Cooked vegetable intake | rs10161952 | C | A | 32.289 | 3.95E-05 | -0.0096 | 0.0017 | 1.30E-08 | -0.0011 | 0.0197 | 9.54E-01 |
| Cooked vegetable intake | rs11138705 | C | G | 32.119 | 3.96E-05 | 0.0104 | 0.0018 | 1.40E-08 | -0.0012 | 0.0212 | 9.56E-01 |
| Cooked vegetable intake | rs12550717 | A | G | 32.204 | 3.95E-05 | 0.0092 | 0.0016 | 1.40E-08 | -0.0240 | 0.0187 | 1.99E-01 |
| Cooked vegetable intake | rs12629972 | C | T | 55.018 | 6.74E-05 | 0.0118 | 0.0016 | 1.20E-13 | 0.0222 | 0.0184 | 2.29E-01 |
| Cooked vegetable intake | rs1421085 | C | T | 42.194 | 5.14E-05 | 0.0103 | 0.0016 | 8.30E-11 | -0.0017 | 0.0184 | 9.27E-01 |
| Cooked vegetable intake | rs1816263 | C | T | 30.299 | 3.70E-05 | 0.0096 | 0.0017 | 3.70E-08 | -0.0318 | 0.0207 | 1.24E-01 |
| Cooked vegetable intake | rs2052063 | T | C | 36.385 | 4.47E-05 | -0.0095 | 0.0016 | 1.60E-09 | 0.0044 | 0.0181 | 8.08E-01 |
| Cooked vegetable intake | rs2102738 | C | A | 34.066 | 4.22E-05 | -0.0122 | 0.0021 | 5.30E-09 | 0.0279 | 0.0239 | 2.44E-01 |
| Cooked vegetable intake | rs2252508 | G | A | 33.949 | 4.14E-05 | 0.0091 | 0.0016 | 5.70E-09 | 0.0180 | 0.0180 | 3.18E-01 |
| Cooked vegetable intake | rs2844672 | A | G | 35.901 | 4.36E-05 | -0.0096 | 0.0016 | 2.10E-09 | 0.0126 | 0.0188 | 5.01E-01 |
| Cooked vegetable intake | rs28450747 | A | G | 30.011 | 3.69E-05 | -0.0102 | 0.0019 | 4.30E-08 | -0.0106 | 0.0219 | 6.28E-01 |
| Cooked vegetable intake | rs28711392 | C | T | 43.345 | 5.37E-05 | -0.0107 | 0.0016 | 4.60E-11 | 0.0215 | 0.0186 | 2.48E-01 |
| Cooked vegetable intake | rs34155012 | T | C | 30.175 | 3.92E-05 | 0.0106 | 0.0019 | 3.90E-08 | 0.0357 | 0.0271 | 1.88E-01 |
| Cooked vegetable intake | rs349062 | C | G | 31.060 | 3.78E-05 | -0.0089 | 0.0016 | 2.50E-08 | -0.0070 | 0.0183 | 7.00E-01 |
| Cooked vegetable intake | rs4851029 | G | T | 42.307 | 5.16E-05 | 0.0102 | 0.0016 | 7.80E-11 | 0.0114 | 0.0182 | 5.30E-01 |
| Cooked vegetable intake | rs838133 | G | A | 52.396 | 6.76E-05 | 0.0117 | 0.0016 | 4.50E-13 | -0.0312 | 0.0200 | 1.19E-01 |
| Tea intake | rs10741694 | C | T | 46.784 | 1.05E-04 | 0.0150 | 0.0022 | 7.90E-12 | 0.0072 | 0.0185 | 6.96E-01 |
| Tea intake | rs10752269 | A | G | 36.878 | 8.28E-05 | -0.0129 | 0.0021 | 1.30E-09 | 0.0293 | 0.0181 | 1.04E-01 |
| Tea intake | rs10764990 | A | G | 31.589 | 7.09E-05 | -0.0122 | 0.0022 | 1.90E-08 | -0.0013 | 0.0193 | 9.46E-01 |
| Tea intake | rs11164870 | G | C | 30.037 | 6.84E-05 | -0.0120 | 0.0022 | 4.20E-08 | -0.0366 | 0.0190 | 5.39E-02 |
| Tea intake | rs1156588 | G | A | 35.241 | 7.93E-05 | -0.0155 | 0.0026 | 2.90E-09 | -0.0052 | 0.0220 | 8.15E-01 |
| Tea intake | rs11587444 | G | A | 41.789 | 9.40E-05 | 0.0140 | 0.0022 | 1.00E-10 | 0.0007 | 0.0183 | 9.70E-01 |
| Tea intake | rs12591786 | T | C | 39.274 | 9.08E-05 | -0.0184 | 0.0029 | 3.70E-10 | -0.0311 | 0.0268 | 2.44E-01 |
| Tea intake | rs13282783 | T | C | 33.289 | 7.53E-05 | -0.0136 | 0.0024 | 7.90E-09 | 0.0013 | 0.0211 | 9.52E-01 |
| Tea intake | rs132904 | C | G | 42.296 | 9.50E-05 | 0.0166 | 0.0026 | 7.80E-11 | 0.0227 | 0.0218 | 2.96E-01 |
| Tea intake | rs141071726 | A | G | 35.754 | 8.63E-05 | 0.0407 | 0.0068 | 2.20E-09 | 0.0055 | 0.0685 | 9.36E-01 |
| Tea intake | rs1453548 | A | T | 35.168 | 7.93E-05 | -0.0133 | 0.0022 | 3.00E-09 | 0.0183 | 0.0193 | 3.43E-01 |
| Tea intake | rs1481012 | G | A | 61.148 | 1.37E-04 | -0.0262 | 0.0034 | 5.30E-15 | 0.0072 | 0.0297 | 8.09E-01 |
| Tea intake | rs149805207 | G | A | 32.685 | 8.76E-05 | -0.0719 | 0.0126 | 1.10E-08 | 0.0474 | 0.1174 | 6.86E-01 |
| Tea intake | rs17245213 | A | G | 31.521 | 7.07E-05 | -0.0146 | 0.0026 | 2.00E-08 | 0.0354 | 0.0223 | 1.12E-01 |
| Tea intake | rs17576658 | A | G | 30.117 | 6.76E-05 | -0.0135 | 0.0025 | 4.10E-08 | 0.0366 | 0.0216 | 9.02E-02 |
| Tea intake | rs17685 | A | G | 95.364 | 2.13E-04 | 0.0231 | 0.0024 | 1.60E-22 | -0.0043 | 0.0202 | 8.31E-01 |
| Tea intake | rs2117137 | G | A | 36.338 | 8.14E-05 | 0.0130 | 0.0022 | 1.70E-09 | -0.0022 | 0.0184 | 9.04E-01 |
| Tea intake | rs2273447 | T | A | 43.991 | 9.91E-05 | 0.0175 | 0.0026 | 3.30E-11 | -0.0073 | 0.0225 | 7.47E-01 |
| Tea intake | rs2279844 | A | G | 30.151 | 6.77E-05 | -0.0120 | 0.0022 | 4.00E-08 | -0.0027 | 0.0184 | 8.84E-01 |
| Tea intake | rs2351187 | A | G | 31.959 | 7.23E-05 | 0.0129 | 0.0023 | 1.60E-08 | -0.0239 | 0.0197 | 2.24E-01 |
| Tea intake | rs2472297 | T | C | 493.646 | 1.10E-03 | 0.0533 | 0.0024 | 2.30E-109 | -0.0143 | 0.0235 | 5.42E-01 |
| Tea intake | rs2478875 | G | A | 70.299 | 1.58E-04 | 0.0219 | 0.0026 | 5.10E-17 | -0.0297 | 0.0218 | 1.74E-01 |
| Tea intake | rs2645929 | G | A | 30.424 | 6.83E-05 | -0.0150 | 0.0027 | 3.50E-08 | 0.0014 | 0.0233 | 9.53E-01 |
| Tea intake | rs34619 | A | G | 30.021 | 6.73E-05 | 0.0117 | 0.0021 | 4.30E-08 | -0.0146 | 0.0182 | 4.23E-01 |
| Tea intake | rs4410790 | C | T | 341.270 | 7.66E-04 | 0.0406 | 0.0022 | 3.40E-76 | 0.0118 | 0.0187 | 5.27E-01 |
| Tea intake | rs4808193 | C | T | 45.240 | 1.02E-04 | 0.0151 | 0.0022 | 1.70E-11 | -0.0183 | 0.0192 | 3.39E-01 |
| Tea intake | rs4817505 | C | T | 48.012 | 1.08E-04 | 0.0151 | 0.0022 | 4.20E-12 | 0.0363 | 0.0187 | 5.19E-02 |
| Tea intake | rs56188862 | C | T | 52.497 | 1.18E-04 | -0.0158 | 0.0022 | 4.30E-13 | -0.0264 | 0.0186 | 1.55E-01 |
| Tea intake | rs56348300 | G | C | 33.799 | 7.59E-05 | 0.0159 | 0.0027 | 6.10E-09 | -0.0202 | 0.0235 | 3.90E-01 |
| Tea intake | rs57462170 | A | G | 31.620 | 7.11E-05 | 0.0192 | 0.0034 | 1.90E-08 | 0.0055 | 0.0300 | 8.55E-01 |
| Tea intake | rs57631352 | G | A | 31.868 | 7.17E-05 | -0.0131 | 0.0023 | 1.70E-08 | 0.0420 | 0.0202 | 3.77E-02 |
| Tea intake | rs6829 | T | C | 30.282 | 6.84E-05 | -0.0119 | 0.0022 | 3.70E-08 | 0.0118 | 0.0185 | 5.23E-01 |
| Tea intake | rs713598 | G | C | 38.590 | 8.63E-05 | 0.0134 | 0.0022 | 5.20E-10 | -0.0268 | 0.0192 | 1.62E-01 |
| Tea intake | rs72797284 | G | A | 51.558 | 1.16E-04 | -0.0171 | 0.0024 | 7.00E-13 | 0.0049 | 0.0205 | 8.10E-01 |
| Tea intake | rs7757102 | G | A | 30.624 | 6.88E-05 | -0.0118 | 0.0021 | 3.10E-08 | -0.0110 | 0.0181 | 5.45E-01 |
| Tea intake | rs9302428 | G | C | 30.949 | 6.94E-05 | 0.0122 | 0.0022 | 2.60E-08 | -0.0013 | 0.0184 | 9.44E-01 |
| Tea intake | rs9624470 | A | G | 136.840 | 3.10E-04 | 0.0252 | 0.0022 | 1.30E-31 | -0.0214 | 0.0184 | 2.45E-01 |
| Tea intake | rs9648476 | A | G | 32.722 | 7.34E-05 | 0.0125 | 0.0022 | 1.10E-08 | -0.0028 | 0.0186 | 8.81E-01 |
| Tea intake | rs977474 | T | C | 58.180 | 1.32E-04 | 0.0218 | 0.0029 | 2.40E-14 | -0.0370 | 0.0257 | 1.50E-01 |
| Tea intake | rs9937354 | A | G | 43.231 | 9.70E-05 | -0.0141 | 0.0021 | 4.90E-11 | -0.0037 | 0.0183 | 8.38E-01 |
| Fresh fruit intake | rs10064431 | C | T | 38.311 | 2.86E-05 | -0.0076 | 0.0012 | 6.00E-10 | -0.0197 | 0.0189 | 2.98E-01 |
| Fresh fruit intake | rs10192394 | T | C | 38.875 | 2.92E-05 | -0.0077 | 0.0012 | 4.50E-10 | -0.0079 | 0.0181 | 6.63E-01 |
| Fresh fruit intake | rs10249294 | A | G | 239.940 | 1.79E-04 | 0.0196 | 0.0013 | 4.10E-54 | -0.0136 | 0.0190 | 4.74E-01 |
| Fresh fruit intake | rs10271924 | T | C | 31.481 | 2.48E-05 | -0.0070 | 0.0013 | 2.00E-08 | 0.0178 | 0.0202 | 3.77E-01 |
| Fresh fruit intake | rs1051547 | C | T | 37.224 | 2.83E-05 | -0.0076 | 0.0012 | 1.10E-09 | -0.0132 | 0.0193 | 4.93E-01 |
| Fresh fruit intake | rs10828266 | G | A | 83.023 | 6.22E-05 | 0.0124 | 0.0014 | 8.10E-20 | -0.0405 | 0.0202 | 4.51E-02 |
| Fresh fruit intake | rs10838724 | T | G | 49.344 | 3.78E-05 | 0.0090 | 0.0013 | 2.10E-12 | -0.0140 | 0.0191 | 4.64E-01 |
| Fresh fruit intake | rs10840126 | G | A | 36.028 | 2.80E-05 | -0.0077 | 0.0013 | 1.90E-09 | -0.0134 | 0.0195 | 4.92E-01 |
| Fresh fruit intake | rs11032362 | A | G | 34.063 | 2.54E-05 | 0.0124 | 0.0021 | 5.30E-09 | -0.0294 | 0.0308 | 3.40E-01 |
| Fresh fruit intake | rs11085749 | A | G | 37.987 | 2.84E-05 | -0.0077 | 0.0013 | 7.10E-10 | -0.0063 | 0.0187 | 7.37E-01 |
| Fresh fruit intake | rs11248509 | T | A | 33.414 | 2.51E-05 | 0.0073 | 0.0013 | 7.40E-09 | -0.0175 | 0.0189 | 3.57E-01 |
| Fresh fruit intake | rs11896330 | A | G | 43.919 | 3.31E-05 | -0.0084 | 0.0013 | 3.40E-11 | 0.0218 | 0.0187 | 2.45E-01 |
| Fresh fruit intake | rs12044599 | G | A | 39.272 | 2.95E-05 | 0.0094 | 0.0015 | 3.70E-10 | -0.0171 | 0.0219 | 4.37E-01 |
| Fresh fruit intake | rs12536253 | C | G | 33.205 | 2.49E-05 | -0.0082 | 0.0014 | 8.30E-09 | -0.0427 | 0.0217 | 4.94E-02 |
| Fresh fruit intake | rs12641371 | T | C | 41.148 | 3.07E-05 | 0.0079 | 0.0012 | 1.40E-10 | -0.0134 | 0.0181 | 4.59E-01 |
| Fresh fruit intake | rs12780952 | A | G | 30.492 | 2.28E-05 | 0.0075 | 0.0014 | 3.40E-08 | -0.0001 | 0.0200 | 9.95E-01 |
| Fresh fruit intake | rs12885598 | A | G | 36.301 | 2.72E-05 | 0.0075 | 0.0012 | 1.70E-09 | -0.0016 | 0.0191 | 9.35E-01 |
| Fresh fruit intake | rs13072255 | C | A | 53.867 | 4.03E-05 | 0.0090 | 0.0012 | 2.10E-13 | -0.0171 | 0.0179 | 3.37E-01 |
| Fresh fruit intake | rs1356292 | T | C | 34.891 | 2.62E-05 | 0.0092 | 0.0016 | 3.50E-09 | -0.0213 | 0.0232 | 3.59E-01 |
| Fresh fruit intake | rs1375566 | A | G | 38.290 | 2.87E-05 | -0.0078 | 0.0013 | 6.10E-10 | 0.0357 | 0.0187 | 5.65E-02 |
| Fresh fruit intake | rs139042899 | C | A | 35.037 | 3.44E-05 | 0.0360 | 0.0061 | 3.20E-09 | -0.2029 | 0.1354 | 1.34E-01 |
| Fresh fruit intake | rs149449 | A | G | 35.656 | 2.65E-05 | 0.0073 | 0.0012 | 2.40E-09 | -0.0127 | 0.0180 | 4.78E-01 |
| Fresh fruit intake | rs1620977 | G | A | 91.453 | 6.83E-05 | -0.0132 | 0.0014 | 1.10E-21 | -0.0089 | 0.0211 | 6.72E-01 |
| Fresh fruit intake | rs17049185 | T | G | 33.444 | 2.54E-05 | 0.0080 | 0.0014 | 7.30E-09 | -0.0062 | 0.0202 | 7.58E-01 |
| Fresh fruit intake | rs1866823 | A | G | 35.876 | 2.74E-05 | 0.0074 | 0.0012 | 2.10E-09 | -0.0186 | 0.0183 | 3.11E-01 |
| Fresh fruit intake | rs2093654 | G | A | 32.078 | 2.41E-05 | 0.0071 | 0.0013 | 1.50E-08 | 0.0115 | 0.0184 | 5.33E-01 |
| Fresh fruit intake | rs2143081 | A | G | 45.844 | 3.44E-05 | 0.0083 | 0.0012 | 1.30E-11 | -0.0130 | 0.0181 | 4.72E-01 |
| Fresh fruit intake | rs2790688 | T | C | 45.544 | 3.42E-05 | 0.0114 | 0.0017 | 1.50E-11 | 0.0118 | 0.0258 | 6.48E-01 |
| Fresh fruit intake | rs28479795 | T | C | 58.113 | 4.35E-05 | 0.0112 | 0.0015 | 2.50E-14 | -0.0123 | 0.0224 | 5.84E-01 |
| Fresh fruit intake | rs2867113 | A | G | 49.997 | 4.37E-05 | -0.0139 | 0.0020 | 1.50E-12 | -0.0397 | 0.0291 | 1.73E-01 |
| Fresh fruit intake | rs329274 | G | A | 30.822 | 2.32E-05 | 0.0068 | 0.0012 | 2.80E-08 | 0.0045 | 0.0183 | 8.05E-01 |
| Fresh fruit intake | rs34162196 | T | C | 79.890 | 5.97E-05 | -0.0181 | 0.0020 | 4.00E-19 | -0.0672 | 0.0325 | 3.89E-02 |
| Fresh fruit intake | rs4302893 | A | G | 32.323 | 2.43E-05 | 0.0074 | 0.0013 | 1.30E-08 | -0.0175 | 0.0189 | 3.52E-01 |
| Fresh fruit intake | rs4953150 | T | C | 42.621 | 3.21E-05 | -0.0084 | 0.0013 | 6.60E-11 | 0.0005 | 0.0191 | 9.78E-01 |
| Fresh fruit intake | rs559734 | C | G | 32.587 | 2.48E-05 | 0.0078 | 0.0014 | 1.10E-08 | -0.0148 | 0.0206 | 4.72E-01 |
| Fresh fruit intake | rs586346 | C | T | 29.900 | 2.22E-05 | -0.0069 | 0.0013 | 4.50E-08 | -0.0086 | 0.0197 | 6.62E-01 |
| Fresh fruit intake | rs60452247 | A | G | 39.422 | 2.94E-05 | 0.0080 | 0.0013 | 3.40E-10 | 0.0031 | 0.0190 | 8.69E-01 |
| Fresh fruit intake | rs6475724 | T | C | 31.580 | 2.37E-05 | 0.0077 | 0.0014 | 1.90E-08 | 0.0200 | 0.0206 | 3.31E-01 |
| Fresh fruit intake | rs72974263 | T | C | 31.679 | 2.37E-05 | 0.0074 | 0.0013 | 1.80E-08 | 0.0273 | 0.0192 | 1.55E-01 |
| Fresh fruit intake | rs73455661 | G | A | 57.104 | 4.27E-05 | 0.0103 | 0.0014 | 4.10E-14 | -0.0333 | 0.0202 | 9.95E-02 |
| Fresh fruit intake | rs739320 | C | T | 49.635 | 3.87E-05 | -0.0090 | 0.0013 | 1.90E-12 | -0.0178 | 0.0217 | 4.12E-01 |
| Fresh fruit intake | rs7554485 | C | T | 40.793 | 3.05E-05 | -0.0080 | 0.0013 | 1.70E-10 | 0.0336 | 0.0185 | 6.92E-02 |
| Fresh fruit intake | rs7818437 | C | T | 30.701 | 2.34E-05 | -0.0080 | 0.0015 | 3.00E-08 | 0.0527 | 0.0216 | 1.46E-02 |
| Fresh fruit intake | rs7869969 | G | A | 33.927 | 2.54E-05 | 0.0076 | 0.0013 | 5.70E-09 | -0.0183 | 0.0189 | 3.34E-01 |
| Fresh fruit intake | rs7982441 | C | T | 37.371 | 2.78E-05 | -0.0084 | 0.0014 | 9.80E-10 | 0.0270 | 0.0205 | 1.88E-01 |
| Fresh fruit intake | rs8095324 | G | A | 30.927 | 2.32E-05 | -0.0069 | 0.0012 | 2.70E-08 | -0.0250 | 0.0184 | 1.76E-01 |
| Fresh fruit intake | rs817223 | C | T | 35.304 | 2.64E-05 | -0.0073 | 0.0012 | 2.80E-09 | -0.0062 | 0.0181 | 7.30E-01 |
| Fresh fruit intake | rs862227 | G | A | 68.703 | 5.11E-05 | -0.0101 | 0.0012 | 1.10E-16 | 0.0030 | 0.0185 | 8.70E-01 |
| Fresh fruit intake | rs9517948 | T | C | 31.807 | 2.39E-05 | 0.0070 | 0.0012 | 1.70E-08 | -0.0235 | 0.0181 | 1.94E-01 |
| Fresh fruit intake | rs9919429 | G | A | 30.226 | 2.26E-05 | -0.0067 | 0.0012 | 3.80E-08 | 0.0077 | 0.0182 | 6.72E-01 |
| Fresh fruit intake | rs994270 | G | C | 84.318 | 6.32E-05 | 0.0133 | 0.0014 | 4.20E-20 | -0.0262 | 0.0209 | 2.11E-01 |
| Cereal intake | rs10057775 | C | T | 47.907 | 7.63E-05 | 0.0200 | 0.0029 | 4.50E-12 | -0.0580 | 0.0303 | 5.57E-02 |
| Cereal intake | rs10857964 | C | T | 40.802 | 6.47E-05 | 0.0141 | 0.0022 | 1.70E-10 | -0.0068 | 0.0227 | 7.66E-01 |
| Cereal intake | rs11038810 | G | A | 35.709 | 5.68E-05 | 0.0111 | 0.0019 | 2.30E-09 | 0.0169 | 0.0188 | 3.69E-01 |
| Cereal intake | rs11097340 | T | C | 40.379 | 6.38E-05 | -0.0115 | 0.0018 | 2.10E-10 | -0.0030 | 0.0183 | 8.71E-01 |
| Cereal intake | rs112780312 | A | G | 36.208 | 5.88E-05 | -0.0121 | 0.0020 | 1.80E-09 | 0.0373 | 0.0207 | 7.17E-02 |
| Cereal intake | rs11670024 | G | A | 32.712 | 5.24E-05 | 0.0160 | 0.0028 | 1.10E-08 | 0.0010 | 0.0281 | 9.72E-01 |
| Cereal intake | rs11940694 | G | A | 47.673 | 7.67E-05 | -0.0127 | 0.0018 | 5.00E-12 | 0.0169 | 0.0184 | 3.58E-01 |
| Cereal intake | rs12354267 | C | T | 36.315 | 5.79E-05 | 0.0116 | 0.0019 | 1.70E-09 | 0.0036 | 0.0195 | 8.52E-01 |
| Cereal intake | rs13234131 | G | A | 40.895 | 6.48E-05 | 0.0170 | 0.0027 | 1.60E-10 | 0.0329 | 0.0277 | 2.34E-01 |
| Cereal intake | rs184643 | A | G | 45.438 | 7.27E-05 | -0.0122 | 0.0018 | 1.60E-11 | 0.0105 | 0.0187 | 5.76E-01 |
| Cereal intake | rs1853931 | A | G | 39.223 | 6.40E-05 | -0.0113 | 0.0018 | 3.80E-10 | -0.0082 | 0.0186 | 6.61E-01 |
| Cereal intake | rs2450126 | G | A | 36.794 | 5.87E-05 | -0.0149 | 0.0025 | 1.30E-09 | -0.0095 | 0.0260 | 7.15E-01 |
| Cereal intake | rs2472297 | T | C | 61.467 | 9.70E-05 | -0.0159 | 0.0020 | 4.50E-15 | -0.0143 | 0.0235 | 5.42E-01 |
| Cereal intake | rs2504706 | C | T | 74.771 | 1.19E-04 | 0.0182 | 0.0021 | 5.30E-18 | -0.0308 | 0.0209 | 1.40E-01 |
| Cereal intake | rs2799849 | T | C | 41.853 | 6.64E-05 | -0.0123 | 0.0019 | 9.80E-11 | 0.0007 | 0.0194 | 9.73E-01 |
| Cereal intake | rs2817377 | A | G | 30.617 | 4.87E-05 | 0.0099 | 0.0018 | 3.10E-08 | -0.0141 | 0.0181 | 4.37E-01 |
| Cereal intake | rs2927238 | G | T | 31.392 | 4.98E-05 | 0.0102 | 0.0018 | 2.10E-08 | 0.0064 | 0.0185 | 7.28E-01 |
| Cereal intake | rs3115230 | A | C | 30.707 | 4.91E-05 | -0.0115 | 0.0021 | 3.00E-08 | 0.0252 | 0.0207 | 2.23E-01 |
| Cereal intake | rs4410790 | C | T | 34.920 | 5.55E-05 | -0.0109 | 0.0018 | 3.40E-09 | 0.0118 | 0.0187 | 5.27E-01 |
| Cereal intake | rs4739095 | A | G | 37.347 | 5.94E-05 | -0.0129 | 0.0021 | 9.90E-10 | 0.0143 | 0.0212 | 5.01E-01 |
| Cereal intake | rs4797242 | A | C | 34.404 | 5.46E-05 | 0.0114 | 0.0019 | 4.50E-09 | 0.0410 | 0.0199 | 3.97E-02 |
| Cereal intake | rs491711 | C | A | 36.539 | 5.87E-05 | 0.0117 | 0.0019 | 1.50E-09 | -0.0164 | 0.0195 | 4.02E-01 |
| Cereal intake | rs4988235 | A | G | 32.091 | 5.04E-05 | 0.0114 | 0.0020 | 1.50E-08 | -0.0371 | 0.0204 | 6.94E-02 |
| Cereal intake | rs56131196 | A | G | 62.511 | 9.92E-05 | 0.0180 | 0.0023 | 2.70E-15 | -0.0009 | 0.0262 | 9.73E-01 |
| Cereal intake | rs62442924 | T | C | 31.814 | 5.07E-05 | 0.0127 | 0.0023 | 1.70E-08 | -0.0212 | 0.0222 | 3.39E-01 |
| Cereal intake | rs6510177 | C | T | 32.475 | 5.32E-05 | -0.0130 | 0.0023 | 1.20E-08 | 0.0657 | 0.0262 | 1.21E-02 |
| Cereal intake | rs6545770 | T | A | 44.392 | 7.10E-05 | -0.0137 | 0.0021 | 2.70E-11 | 0.0185 | 0.0211 | 3.79E-01 |
| Cereal intake | rs67723420 | A | T | 32.486 | 5.20E-05 | 0.0105 | 0.0018 | 1.20E-08 | -0.0243 | 0.0189 | 1.98E-01 |
| Cereal intake | rs68136852 | A | C | 32.466 | 5.15E-05 | -0.0141 | 0.0025 | 1.20E-08 | 0.0546 | 0.0254 | 3.16E-02 |
| Cereal intake | rs6918737 | A | T | 42.368 | 6.77E-05 | 0.0137 | 0.0021 | 7.60E-11 | -0.0155 | 0.0215 | 4.69E-01 |
| Cereal intake | rs7040561 | A | T | 41.676 | 6.73E-05 | -0.0163 | 0.0025 | 1.10E-10 | 0.0548 | 0.0275 | 4.65E-02 |
| Cereal intake | rs7619139 | A | T | 87.225 | 1.39E-04 | -0.0170 | 0.0018 | 9.70E-21 | 0.0211 | 0.0184 | 2.51E-01 |
| Cereal intake | rs78854891 | C | T | 37.140 | 6.02E-05 | 0.0221 | 0.0036 | 1.10E-09 | 0.0060 | 0.0433 | 8.90E-01 |
| Cereal intake | rs79642906 | A | G | 31.640 | 5.04E-05 | -0.0182 | 0.0032 | 1.90E-08 | 0.0502 | 0.0319 | 1.15E-01 |
| Cereal intake | rs8097544 | G | A | 94.001 | 1.51E-04 | -0.0246 | 0.0025 | 3.20E-22 | 0.0410 | 0.0254 | 1.06E-01 |
| Cereal intake | rs838133 | G | A | 125.518 | 2.11E-04 | -0.0207 | 0.0018 | 3.90E-29 | -0.0312 | 0.0200 | 1.19E-01 |
| Cereal intake | rs9374896 | T | C | 95.694 | 1.53E-04 | 0.0175 | 0.0018 | 1.30E-22 | -0.0335 | 0.0183 | 6.72E-02 |
| Cereal intake | rs9846396 | T | C | 44.188 | 7.06E-05 | 0.0120 | 0.0018 | 3.00E-11 | -0.0246 | 0.0183 | 1.77E-01 |
| Cereal intake | rs9987289 | G | A | 33.336 | 5.29E-05 | 0.0179 | 0.0031 | 7.80E-09 | 0.0530 | 0.0323 | 1.01E-01 |
| Salad / raw vegetable intake | rs1052352 | T | C | 32.797 | 3.33E-05 | 0.0082 | 0.0014 | 1.00E-08 | -0.0532 | 0.0183 | 3.63E-03 |
| Salad / raw vegetable intake | rs10819082 | A | G | 36.671 | 3.73E-05 | -0.0092 | 0.0015 | 1.40E-09 | 0.0238 | 0.0190 | 2.11E-01 |
| Salad / raw vegetable intake | rs12203592 | T | C | 36.866 | 3.62E-05 | -0.0103 | 0.0017 | 1.30E-09 | -0.0124 | 0.0286 | 6.64E-01 |
| Salad / raw vegetable intake | rs12908495 | A | C | 31.508 | 3.21E-05 | -0.0094 | 0.0017 | 2.00E-08 | 0.0111 | 0.0210 | 5.95E-01 |
| Salad / raw vegetable intake | rs17460017 | T | A | 37.973 | 3.85E-05 | 0.0112 | 0.0018 | 7.20E-10 | -0.0156 | 0.0232 | 5.01E-01 |
| Salad / raw vegetable intake | rs1890012 | G | T | 33.255 | 3.41E-05 | -0.0104 | 0.0018 | 8.10E-09 | -0.0129 | 0.0227 | 5.71E-01 |
| Salad / raw vegetable intake | rs3095337 | C | G | 51.060 | 5.17E-05 | -0.0126 | 0.0018 | 9.00E-13 | 0.0580 | 0.0236 | 1.40E-02 |
| Salad / raw vegetable intake | rs34186148 | C | G | 29.800 | 3.02E-05 | -0.0081 | 0.0015 | 4.80E-08 | 0.0034 | 0.0187 | 8.55E-01 |
| Salad / raw vegetable intake | rs4083969 | G | C | 30.248 | 3.17E-05 | 0.0171 | 0.0031 | 3.80E-08 | -0.0541 | 0.0409 | 1.86E-01 |
| Salad / raw vegetable intake | rs4291983 | A | C | 34.756 | 3.52E-05 | -0.0084 | 0.0014 | 3.70E-09 | -0.0100 | 0.0179 | 5.77E-01 |
| Salad / raw vegetable intake | rs57221424 | G | C | 34.004 | 3.49E-05 | 0.0089 | 0.0015 | 5.50E-09 | -0.0199 | 0.0201 | 3.24E-01 |
| Salad / raw vegetable intake | rs62461186 | C | A | 37.303 | 3.80E-05 | -0.0113 | 0.0019 | 1.00E-09 | 0.0034 | 0.0235 | 8.87E-01 |
| Salad / raw vegetable intake | rs6482190 | G | A | 50.242 | 5.13E-05 | 0.0113 | 0.0016 | 1.40E-12 | -0.0373 | 0.0210 | 7.51E-02 |
| Salad / raw vegetable intake | rs75248709 | T | C | 31.292 | 3.41E-05 | -0.0197 | 0.0035 | 2.20E-08 | -0.0103 | 0.0487 | 8.32E-01 |
| Salad / raw vegetable intake | rs7619139 | A | T | 73.949 | 7.54E-05 | 0.0125 | 0.0015 | 8.00E-18 | 0.0211 | 0.0184 | 2.51E-01 |
| Salad / raw vegetable intake | rs7821179 | C | G | 29.961 | 3.04E-05 | -0.0108 | 0.0020 | 4.40E-08 | -0.0274 | 0.0249 | 2.71E-01 |
| Salad / raw vegetable intake | rs790561 | G | A | 63.776 | 6.48E-05 | 0.0125 | 0.0016 | 1.40E-15 | 0.0118 | 0.0197 | 5.51E-01 |
| Salad / raw vegetable intake | rs8130508 | A | G | 30.681 | 3.14E-05 | 0.0087 | 0.0016 | 3.00E-08 | -0.0194 | 0.0200 | 3.32E-01 |
| Coffee intake | rs1057868 | T | C | 124.901 | 1.62E-04 | 0.0200 | 0.0018 | 5.40E-29 | -0.0055 | 0.0200 | 7.84E-01 |
| Coffee intake | rs117810762 | A | G | 33.775 | 4.53E-05 | 0.0359 | 0.0062 | 6.20E-09 | 0.0094 | 0.0691 | 8.92E-01 |
| Coffee intake | rs117968677 | A | G | 31.645 | 4.55E-05 | -0.0310 | 0.0055 | 1.90E-08 | 0.1162 | 0.0696 | 9.50E-02 |
| Coffee intake | rs12514566 | A | G | 44.651 | 5.81E-05 | -0.0114 | 0.0017 | 2.40E-11 | 0.0458 | 0.0191 | 1.64E-02 |
| Coffee intake | rs12989746 | T | G | 30.821 | 4.02E-05 | 0.0104 | 0.0019 | 2.80E-08 | -0.0369 | 0.0207 | 7.50E-02 |
| Coffee intake | rs13054099 | C | T | 34.460 | 4.48E-05 | -0.0108 | 0.0018 | 4.30E-09 | -0.0022 | 0.0209 | 9.16E-01 |
| Coffee intake | rs13163336 | A | C | 45.742 | 5.93E-05 | 0.0149 | 0.0022 | 1.30E-11 | 0.0017 | 0.0243 | 9.44E-01 |
| Coffee intake | rs1338549 | G | T | 33.961 | 4.45E-05 | -0.0095 | 0.0016 | 5.60E-09 | 0.0334 | 0.0183 | 6.77E-02 |
| Coffee intake | rs13387939 | A | C | 59.930 | 7.79E-05 | 0.0166 | 0.0021 | 9.80E-15 | 0.0341 | 0.0236 | 1.48E-01 |
| Coffee intake | rs1421085 | C | T | 127.159 | 1.66E-04 | 0.0185 | 0.0016 | 1.70E-29 | -0.0017 | 0.0184 | 9.27E-01 |
| Coffee intake | rs1527961 | C | T | 31.808 | 4.16E-05 | -0.0133 | 0.0024 | 1.70E-08 | 0.0115 | 0.0258 | 6.56E-01 |
| Coffee intake | rs17842490 | G | A | 44.012 | 5.73E-05 | -0.0452 | 0.0068 | 3.30E-11 | 0.0786 | 0.0736 | 2.86E-01 |
| Coffee intake | rs1942965 | C | T | 30.236 | 3.96E-05 | -0.0089 | 0.0016 | 3.80E-08 | 0.0233 | 0.0181 | 1.99E-01 |
| Coffee intake | rs2189234 | G | T | 36.172 | 4.71E-05 | 0.0100 | 0.0017 | 1.80E-09 | -0.0251 | 0.0186 | 1.77E-01 |
| Coffee intake | rs2465037 | A | C | 38.772 | 5.09E-05 | -0.0106 | 0.0017 | 4.80E-10 | -0.0243 | 0.0188 | 1.95E-01 |
| Coffee intake | rs2472297 | T | C | 646.735 | 8.37E-04 | 0.0465 | 0.0018 | 1.10E-142 | -0.0143 | 0.0235 | 5.42E-01 |
| Coffee intake | rs2597805 | T | C | 31.489 | 4.21E-05 | 0.0099 | 0.0018 | 2.00E-08 | -0.0119 | 0.0198 | 5.48E-01 |
| Coffee intake | rs34060476 | G | A | 60.450 | 7.88E-05 | 0.0184 | 0.0024 | 7.50E-15 | 0.0387 | 0.0274 | 1.58E-01 |
| Coffee intake | rs4410790 | C | T | 545.509 | 7.10E-04 | 0.0391 | 0.0017 | 1.20E-120 | 0.0118 | 0.0187 | 5.27E-01 |
| Coffee intake | rs442355 | C | G | 36.096 | 4.71E-05 | -0.0111 | 0.0019 | 1.90E-09 | -0.0073 | 0.0205 | 7.24E-01 |
| Coffee intake | rs4615895 | A | G | 43.520 | 5.72E-05 | 0.0122 | 0.0018 | 4.20E-11 | -0.0006 | 0.0204 | 9.76E-01 |
| Coffee intake | rs476828 | C | T | 83.751 | 1.09E-04 | 0.0173 | 0.0019 | 5.60E-20 | 0.0075 | 0.0210 | 7.21E-01 |
| Coffee intake | rs516636 | A | C | 34.632 | 4.51E-05 | 0.0117 | 0.0020 | 4.00E-09 | 0.0047 | 0.0229 | 8.37E-01 |
| Coffee intake | rs56113850 | C | T | 60.129 | 7.83E-05 | 0.0127 | 0.0016 | 8.90E-15 | 0.0104 | 0.0200 | 6.03E-01 |
| Coffee intake | rs57918684 | A | G | 33.141 | 4.34E-05 | 0.0129 | 0.0022 | 8.60E-09 | -0.0170 | 0.0250 | 4.97E-01 |
| Coffee intake | rs6062682 | T | C | 40.020 | 5.35E-05 | 0.0104 | 0.0016 | 2.50E-10 | -0.0274 | 0.0181 | 1.29E-01 |
| Coffee intake | rs6063085 | C | A | 38.899 | 5.07E-05 | 0.0104 | 0.0017 | 4.50E-10 | -0.0018 | 0.0187 | 9.21E-01 |
| Coffee intake | rs61928609 | C | A | 45.854 | 5.97E-05 | -0.0147 | 0.0022 | 1.30E-11 | -0.0377 | 0.0258 | 1.44E-01 |
| Coffee intake | rs62064918 | T | C | 30.101 | 3.93E-05 | -0.0103 | 0.0019 | 4.10E-08 | -0.0135 | 0.0214 | 5.28E-01 |
| Coffee intake | rs630194 | C | T | 44.678 | 5.81E-05 | -0.0114 | 0.0017 | 2.30E-11 | -0.0238 | 0.0191 | 2.13E-01 |
| Coffee intake | rs6469262 | C | T | 31.576 | 4.12E-05 | -0.0092 | 0.0016 | 1.90E-08 | -0.0285 | 0.0183 | 1.19E-01 |
| Coffee intake | rs73075167 | T | A | 43.191 | 5.81E-05 | -0.0161 | 0.0024 | 5.00E-11 | 0.0196 | 0.0286 | 4.93E-01 |
| Coffee intake | rs75347775 | A | G | 30.933 | 4.04E-05 | 0.0105 | 0.0019 | 2.70E-08 | -0.0004 | 0.0223 | 9.85E-01 |
| Coffee intake | rs780093 | C | T | 64.366 | 8.36E-05 | 0.0133 | 0.0017 | 1.00E-15 | 0.0423 | 0.0184 | 2.12E-02 |
| Coffee intake | rs7811609 | T | C | 30.137 | 3.91E-05 | 0.0091 | 0.0017 | 4.00E-08 | 0.0085 | 0.0184 | 6.44E-01 |
| Coffee intake | rs78267637 | G | C | 34.695 | 4.74E-05 | -0.0254 | 0.0043 | 3.90E-09 | -0.0302 | 0.0545 | 5.80E-01 |
| Coffee intake | rs8056750 | T | C | 36.776 | 5.11E-05 | 0.0105 | 0.0017 | 1.30E-09 | -0.0001 | 0.0209 | 9.98E-01 |
| Coffee intake | rs9398171 | T | C | 37.210 | 4.85E-05 | 0.0109 | 0.0018 | 1.10E-09 | 0.0178 | 0.0197 | 3.66E-01 |
| Dried fruit intake | rs10026792 | A | G | 34.662 | 4.85E-05 | 0.0108 | 0.0018 | 3.90E-09 | 0.0049 | 0.0197 | 8.04E-01 |
| Dried fruit intake | rs10129747 | G | A | 30.988 | 4.36E-05 | 0.0094 | 0.0017 | 2.60E-08 | -0.0017 | 0.0181 | 9.25E-01 |
| Dried fruit intake | rs10740991 | C | G | 81.233 | 1.14E-04 | 0.0167 | 0.0019 | 2.00E-19 | -0.0412 | 0.0206 | 4.53E-02 |
| Dried fruit intake | rs10896126 | G | A | 68.069 | 9.53E-05 | -0.0150 | 0.0018 | 1.60E-16 | 0.0117 | 0.0197 | 5.53E-01 |
| Dried fruit intake | rs11152349 | A | G | 29.740 | 4.15E-05 | 0.0099 | 0.0018 | 4.90E-08 | 0.0062 | 0.0198 | 7.53E-01 |
| Dried fruit intake | rs11586016 | C | G | 32.590 | 4.55E-05 | 0.0099 | 0.0017 | 1.10E-08 | 0.0268 | 0.0188 | 1.53E-01 |
| Dried fruit intake | rs11632215 | C | A | 29.963 | 4.23E-05 | -0.0141 | 0.0026 | 4.40E-08 | -0.0481 | 0.0301 | 1.10E-01 |
| Dried fruit intake | rs11720884 | G | A | 33.362 | 4.69E-05 | 0.0112 | 0.0019 | 7.60E-09 | -0.0068 | 0.0206 | 7.42E-01 |
| Dried fruit intake | rs11772627 | C | G | 71.316 | 1.00E-04 | 0.0183 | 0.0022 | 3.00E-17 | -0.0089 | 0.0225 | 6.92E-01 |
| Dried fruit intake | rs11811826 | A | T | 43.425 | 6.08E-05 | 0.0132 | 0.0020 | 4.40E-11 | -0.0056 | 0.0216 | 7.95E-01 |
| Dried fruit intake | rs12137234 | T | C | 30.847 | 4.41E-05 | 0.0102 | 0.0018 | 2.80E-08 | 0.0138 | 0.0206 | 5.02E-01 |
| Dried fruit intake | rs1582322 | G | A | 33.588 | 4.73E-05 | 0.0099 | 0.0017 | 6.80E-09 | -0.0174 | 0.0185 | 3.47E-01 |
| Dried fruit intake | rs1622515 | G | A | 35.233 | 4.91E-05 | 0.0099 | 0.0017 | 2.90E-09 | -0.0043 | 0.0188 | 8.19E-01 |
| Dried fruit intake | rs1648404 | T | C | 31.655 | 4.42E-05 | 0.0094 | 0.0017 | 1.80E-08 | 0.0035 | 0.0181 | 8.46E-01 |
| Dried fruit intake | rs17175518 | A | C | 33.878 | 4.72E-05 | 0.0115 | 0.0020 | 5.90E-09 | 0.0037 | 0.0211 | 8.61E-01 |
| Dried fruit intake | rs17184707 | T | C | 31.434 | 4.38E-05 | -0.0114 | 0.0020 | 2.10E-08 | 0.0339 | 0.0226 | 1.34E-01 |
| Dried fruit intake | rs1797235 | C | G | 33.076 | 4.70E-05 | -0.0100 | 0.0017 | 8.90E-09 | -0.0140 | 0.0185 | 4.51E-01 |
| Dried fruit intake | rs2328887 | C | T | 46.588 | 6.49E-05 | 0.0189 | 0.0028 | 8.80E-12 | -0.0075 | 0.0337 | 8.24E-01 |
| Dried fruit intake | rs2533273 | A | C | 34.680 | 4.87E-05 | -0.0099 | 0.0017 | 3.90E-09 | 0.0131 | 0.0194 | 4.98E-01 |
| Dried fruit intake | rs261809 | G | A | 32.886 | 4.60E-05 | -0.0096 | 0.0017 | 9.80E-09 | -0.0047 | 0.0183 | 7.96E-01 |
| Dried fruit intake | rs3101339 | C | A | 69.908 | 9.73E-05 | 0.0143 | 0.0017 | 6.20E-17 | -0.0098 | 0.0185 | 5.99E-01 |
| Dried fruit intake | rs34162196 | T | C | 65.098 | 9.08E-05 | -0.0224 | 0.0028 | 7.10E-16 | -0.0672 | 0.0325 | 3.89E-02 |
| Dried fruit intake | rs3764002 | T | C | 47.653 | 6.65E-05 | 0.0131 | 0.0019 | 5.10E-12 | -0.0145 | 0.0221 | 5.11E-01 |
| Dried fruit intake | rs4140799 | A | G | 31.743 | 4.45E-05 | 0.0095 | 0.0017 | 1.80E-08 | -0.0053 | 0.0181 | 7.69E-01 |
| Dried fruit intake | rs4149513 | A | G | 49.254 | 6.88E-05 | 0.0117 | 0.0017 | 2.20E-12 | -0.0073 | 0.0180 | 6.84E-01 |
| Dried fruit intake | rs4269101 | G | T | 55.173 | 7.71E-05 | -0.0138 | 0.0019 | 1.10E-13 | 0.0085 | 0.0198 | 6.69E-01 |
| Dried fruit intake | rs429358 | C | T | 74.313 | 1.04E-04 | 0.0199 | 0.0023 | 6.70E-18 | -0.0008 | 0.0267 | 9.75E-01 |
| Dried fruit intake | rs4800488 | A | C | 51.368 | 7.18E-05 | 0.0120 | 0.0017 | 7.70E-13 | -0.0148 | 0.0180 | 4.12E-01 |
| Dried fruit intake | rs57499472 | C | T | 33.262 | 4.73E-05 | 0.0099 | 0.0017 | 8.10E-09 | -0.0083 | 0.0184 | 6.52E-01 |
| Dried fruit intake | rs62084586 | C | T | 35.073 | 4.96E-05 | 0.0134 | 0.0023 | 3.20E-09 | 0.0211 | 0.0246 | 3.92E-01 |
| Dried fruit intake | rs72720396 | G | A | 33.123 | 4.61E-05 | 0.0114 | 0.0020 | 8.70E-09 | -0.0266 | 0.0226 | 2.39E-01 |
| Dried fruit intake | rs746868 | G | C | 56.658 | 7.89E-05 | -0.0129 | 0.0017 | 5.20E-14 | 0.0505 | 0.0184 | 5.90E-03 |
| Dried fruit intake | rs75641275 | C | A | 35.250 | 4.93E-05 | -0.0142 | 0.0024 | 2.90E-09 | -0.0180 | 0.0262 | 4.92E-01 |
| Dried fruit intake | rs7582086 | T | G | 33.089 | 4.62E-05 | -0.0096 | 0.0017 | 8.80E-09 | 0.0146 | 0.0180 | 4.17E-01 |
| Dried fruit intake | rs7599488 | T | C | 38.104 | 5.31E-05 | -0.0104 | 0.0017 | 6.70E-10 | 0.0290 | 0.0182 | 1.11E-01 |
| Dried fruit intake | rs7808471 | C | T | 41.720 | 5.81E-05 | -0.0115 | 0.0018 | 1.10E-10 | 0.0120 | 0.0193 | 5.35E-01 |
| Dried fruit intake | rs7829800 | G | A | 34.169 | 4.82E-05 | -0.0104 | 0.0018 | 5.10E-09 | 0.0303 | 0.0191 | 1.12E-01 |
| Dried fruit intake | rs8081370 | T | C | 32.181 | 4.54E-05 | -0.0167 | 0.0029 | 1.40E-08 | 0.0214 | 0.0357 | 5.48E-01 |
| Dried fruit intake | rs862227 | G | A | 30.030 | 4.17E-05 | -0.0092 | 0.0017 | 4.30E-08 | 0.0030 | 0.0185 | 8.70E-01 |
| Dried fruit intake | rs893856 | A | G | 32.346 | 4.53E-05 | -0.0134 | 0.0023 | 1.30E-08 | 0.0308 | 0.0250 | 2.18E-01 |
| Dried fruit intake | rs9385269 | T | C | 51.482 | 7.26E-05 | 0.0121 | 0.0017 | 7.20E-13 | -0.0372 | 0.0181 | 3.93E-02 |

se: standard error, pval: p-value, SNP: single nucleotide polymorphism

**Supplementary Table 3** The result of sensitivity analyses.

| Exposure(Dietary factors) | Heterogeneity Test(Cochrane's Q test) | | | | Pleiotropy Test | | | MR-PRESSO | |
| --- | --- | --- | --- | --- | --- | --- | --- | --- | --- |
|  | IVW | | MR-Egger | | MR- Egger intercept | se | pval | Disortion Test | Global Test |
|  | Q | pval | Q | pval |  |  |  | Outliers | pval |
| Alcoholic drinks per week | 39.178 | 0.149 | 39.043 | 0.125 | 0.002 | 0.007 | 0.748 | NA | 0.186 |
| Processed meat intake | 24.071 | 0.344 | 22.863 | 0.351 | 0.024 | 0.023 | 0.304 | NA | 0.326 |
| Poultry intake | 6.435 | 0.376 | 4.009 | 0.548 | -0.330 | 0.211 | 0.180 | NA | 0.424 |
| Beef intake | 8.492 | 0.862 | 8.426 | 0.815 | -0.007 | 0.029 | 0.800 | NA | 0.851 |
| Non-oily fish intake | 12.050 | 0.282 | 11.670 | 0.233 | 0.018 | 0.034 | 0.601 | NA | 0.333 |
| Oily fish intake | 62.697 | 0.313 | 61.851 | 0.307 | 0.010 | 0.012 | 0.894 | NA | 0.325 |
| Pork intake | 10.327 | 0.667 | 10.326 | 0.587 | 0.001 | 0.038 | 0.984 | NA | 0.660 |
| Lamb/mutton intake | 18.082 | 0.957 | 17.506 | 0.953 | -0.012 | 0.016 | 0.454 | NA | 0.963 |
| Bread intake | 35.342 | 0.160 | 35.058 | 0.137 | 0.010 | 0.021 | 0.644 | NA | 0.147 |
| Cheese intake | 74.221 | 0.120 | 66.672 | 0.258 | -0.031 | 0.012 | 0.011 | NA | 0.115 |
| Cooked vegetable intake | 14.275 | 0.578 | 14.095 | 0.518 | 0.022 | 0.052 | 0.677 | NA | 0.568 |
| Tea intake | 36.537 | 0.583 | 36.219 | 0.552 | -0.004 | 0.008 | 0.576 | NA | 0.590 |
| Fresh fruit intake | 54.003 | 0.324 | 54.001 | 0.289 | -0.000 | 0.011 | 0.964 | NA | 0.328 |
| Cereal intake | 48.436 | 0.120 | 48.412 | 0.099 | -0.002 | 0.017 | 0.894 | NA | 0.123 |
| Salad/raw vegetable intake | 23.825 | 0.124 | 22.882 | 0.117 | -0.025 | 0.031 | 0.428 | NA | 0.124 |
| Coffee intake | 43.828 | 0.204 | 43.801 | 0.174 | -0.001 | 0.008 | 0.880 | NA | 0.232 |
| Dried fruit intake | 36.933 | 0.609 | 36.684 | 0.576 | -0.007 | 0.015 | 0.621 | NA | 0.590 |

se: standard error, pval: p-value, IVW: Inverse variance weighted.
